# Adverse childhood experiences and risk of diabetes: A systematic review and meta-analysis

**DOI:** 10.7189/jogh.12.04082

**Published:** 2022-11-02

**Authors:** Siyu Zhu, Shiyi Shan, Wen Liu, Shuting Li, Leying Hou, Xuanyin Huang, Yi Liu, Qian Yi, Weidi Sun, Kun Tang, Davies Adeloye, Igor Rudan, Peige Song, Global Health Epidemiology Research Group (GHERG)

**Affiliations:** 1 School of Public Health and Women's Hospital, Zhejiang University School of Medicine, Zhejiang University, Zhejiang, China; 2 Vanke School of Public Health, Tsinghua University, Beijing, China; 3 Centre for Global Health Research, University of Edinburgh, Edinburgh, UK

## Abstract

**Background:**

The association between adverse childhood experiences (ACEs) and diabetes is unclear. This systematic review and meta-analysis aims to quantify the association between the number and types of ACEs and diabetes during adulthood based on available observational studies.

**Methods:**

A comprehensive literature search of studies exploring the association between ACEs and diabetes was conducted in PubMed, Medline, and Embase databases until 15 April 2022. A random-effects model was used to pool odds ratios (ORs) and 95% confidence intervals (CIs) for the number and types of ACEs with diabetes. Regarding the association between the number of ACEs and diabetes, we used funnel plots to examine publication bias, subgroup analysis to explore sources of heterogeneity, and sensitivity analysis to explore the robustness of the pooled results.

**Results:**

A total of 49 studies were included. Individuals with higher continuous ACEs (per each additional ACE: OR = 1.06, 95% CI = 1.02-1.10), any ACE (OR = 1.22, 95% CI = 1.16-1.28), or ≥4 ACEs (OR = 1.44, 95% CI = 1.27-1.63) were at an increased risk of diabetes in adulthood when compared with individuals without ACEs. Across specific ACE types, childhood economic adversity (OR = 1.11, 95% CI = 1.04-1.19), physical abuse (OR = 1.14, 95% CI = 1.07-1.21), sexual abuse (OR = 1.25, 95% CI = 1.12-1.39), verbal abuse (OR = 1.11, 95% CI = 1.03-1.20), and incarceration (OR = 1.22, 95% CI = 1.03-1.45) were associated with diabetes. However, neglect, emotional abuse, domestic violence, parental divorce or separation, parental death, and living with a family member with substance abuse or mental disorders were not significantly associated with diabetes.

**Conclusions:**

Individuals with ACEs may have a cumulative risk for diabetes in adulthood. It is critical to prevent ACEs and build resilience in individuals affected by ACEs.

Diabetes has become one of the leading causes of death and disability globally. Type 1 diabetes mellitus (T1DM), type 2 diabetes mellitus (T2DM), and gestational diabetes mellitus (GDM) are the three most common types of diabetes [1]. According to the International Diabetes Federation, there were 537 million adult diabetes cases in 2021 and that number is expected to increase to 783 million by 2045 [2]. Globally, diabetes caused 4.2 to 6.7 million deaths from 2019 to 2021 [2,3]. At the regional level, diabetes has been shown to heighten the risk of mortality. For example, diabetes in the Asia population is associated with a 1.89-fold increase in the risk of death from all-cause mortality [4]. Furthermore, sequential complications such as visual impairment, heart disease, and stroke can lead to disability and a dramatic burden on individuals’ financial status and living quality, especially in settings with poor prognosis and management of diabetes [2].

Hereditary, socioeconomic, and behavioural risk factors, such as infrequent physical exercise and smoking, have often been associated with diabetes [5,6]. However, recent research has revealed that the long-term effects of early life experiences like adverse childhood experiences (ACEs) might also be associated with the development of diabetes [7-9]. ACEs are defined as intensively stressful experiences, such as abuse, neglect, household dysfunction, etc., experienced by individuals aged 18 years or below [10]. There is widespread research interest in the high global prevalence of ACEs [11-13] and their associations with lifelong mental disorders, chronic diseases, and premature mortality [14,15]. For example, one previous meta-analysis revealed that exposure to at least two ACEs was associated with diabetes in adulthood [8] and another found that exposure to at least four ACEs increased the risk of diabetes [7]. A 2015 meta-analysis by Huang et al. [9] found that people suffering neglect, physical abuse, or sexual abuse in childhood had a higher risk of T2DM than those free from these ACEs.

These previous studies have explored the association between either the number or types of ACEs and the risk of diabetes, with findings regarding the association between the number of ACEs and diabetes still inconsistent, and those about the impact of specific types of ACEs on diabetes unclear. To fill up this gap of knowledge, this systematic review and meta-analysis aims to comprehensively assess the associations between the number and types of ACEs and the risk of different types of diabetes (T1DM, T2DM, and GDM).

## METHODS

This study was pre-registered at the International Prospective Registration of Systematic Reviews online protocol (PROSPERO; Registration number: CRD 42022310228) [16], follows the recommendations of the Preferred Reporting Items for Systematic Reviews and Meta-Analyses (PRISMA) [17,18] statement, and refers to the Cochrane Handbook of Systematic Reviews of Interventions [19] for systematic reviews.

### Literature search

Three electronic databases (Medline, PubMed, and Embase) were searched to identify observational studies reporting associations of the number and types of ACEs with diabetes. To reduce heterogeneity introduced by the development of diabetes detection technology in recent years, we only included studies conducted and published in the 21st century. A broad set of search terms was created using a combination of keywords related to ACEs (ACE or ACEs or adverse childhood experience* or adverse childhood event* or childhood adversit*) and diabetes (diabet*) (Table S1 in the **Online Supplementary Document****)**. Reference lists of publications found through the database search were examined for additional relevant articles. The final literature search was conducted on April 15, 2022.

### Study selection

Studies were included in the final analysis if they met the following criteria: 1) the study used an observational design; 2) the exposure was ACE, including the number (eg, one ACE, two ACEs) or types (eg, physical abuse, sexual abuse, neglect) of ACEs; 3) the outcome was diabetes (ie, T1DM, T2DM, or GDM); 4) effect values, including odds ratio (OR), relative risk (RR), or hazard ratio (HR), were provided with confidence intervals (CIs) or relevant data were available to enable calculation for effect values that provide the statistical association between ACEs and diabetes.

Studies were excluded if they were in vitro studies, animal studies, randomized controlled trials, reviews, case reports, and other non-original studies, if they had insufficient data, or if data could not be obtained by contacting corresponding authors. Furthermore, if more than one study reported data from the same sample and outcome, only the one with a larger sample size or better population representativeness was selected.

Two independent authors (SZ and WL) were involved in the study selection. The first round of screening involved an examination of titles and abstracts to exclude studies with irrelevant topics. Next, the remaining articles were rigorously screened in the full text following the inclusion and exclusion criteria. Discrepancies in the review process were solved through discussion or by consulting the senior investigator (PS).

### Variable of interest

#### Adverse childhood experiences

ACEs were defined as exposure to one or more of the following adversities before the age of 18 years: neglect (eg, emotional and physical neglect), abuse (eg, emotional, physical, sexual, and verbal abuse), household dysfunction (eg, domestic violence, parental substance abuse, parental incarceration, parental separation or divorce, parental mental disorder, and parental death), peer violence (eg, bullying), economic adversity, and other types of ACEs [10,20]. Self-reports, clinical interviews, and agency records were all considered for inclusion in studies that used any measurement of ACEs. The approaches used by studies to report ACEs were heterogeneous. To enable comparability between studies, the following scheme was utilized: 1) when the number of ACEs was regarded as a continuous variable, we used the term “continuous ACEs” to measure the influence of per each additional ACE on diabetes; 2) when the number of ACEs was regarded as a binary variable, the term “any ACE” referred to having ever experienced ACEs with no ACEs as the reference, regardless of the number of ACEs; 3) when specifying the number of ACEs, 1 ACE represented suffering only one type of ACE, excluding multiple ACEs; 2 ACEs referred to suffering two types of ACEs, excluding 1 ACE, 3 ACEs, and other multiple ACEs; 3 ACEs referred to suffering three types of ACEs; ≥4 ACEs referred to suffering four or more types of ACEs.

#### Diabetes

The definition of diabetes was based on: 1) self-reported diagnosis of diabetes by a physician or other health professionals; 2) medical records of having been diagnosed with diabetes or treated for diabetes or high blood glucose; 3) reimbursement for diabetes-related medication. T1DM, T2DM, and GDM were all included as outcomes. Throughout this study, we classified outcomes into five categories: diabetes (all types of diabetes), T1DM, T2DM, GDM, and non-GDM (all types of diabetes excluding GDM).

### Data extraction

A standardized data collection form was used for data extraction. Extracted data included: 1) general characteristics of the study (eg, first author, country, publication year, the World Health Organization (WHO) region, and study design); 2) demographics of the study sample (eg, sample size, age, and sex); 3) the number and types of ACEs; 4) assessment of diabetes; 5) effect size (eg, OR, HR, RR, and CI in analyses with the largest adjustment for covariates), and covariates included in the adjustment. Data reported based on subgroups (eg, male and female separately, urban and rural separately) were treated as different data points. Two reviewers (SZ and SS) performed data extraction independently and cross-checked for consistency. Discrepancies were consulted with a senior investigator (PS).

### Study quality assessment

The Newcastle-Ottawa Scale (NOS) [21] was used to assess the quality of cohort studies. The quality criterion was divided into three groups according to the eight NOS items: selection of study groups, comparability of groups, and determination of outcomes. The maximum score for each item was one point, except for comparability, which allowed two points, and the minimum score was zero point. The score range was 0-9 in NOS. To assess cross-sectional studies, the Agency for Healthcare Research and Quality Scale (AHRQ) [22] was used. Each quality criterion with 11 items met by these studies was given one or zero points. The score range was 0-11 in AHRQ. Higher scores indicated better quality of study methodology.

### Statistical analyses

#### Meta-analysis

Subgroup level ORs (eg, sex-specific ORs) within each study were combined with the fixed-effects model before analysis. Heterogeneity among the included studies was assessed using the *I^2^* statistic (significance level set at *I^2^*>50%) and Cochran's *Q* test (significance level set at *P* < 0.10). The degree of heterogeneity across studies was considered as low (*I^2^*<25%), moderate (*I^2^* = 25%-75%), and high (*I^2^*>75%) [23]. To ensure the stability and reliability of the results, a random-effects meta-analysis was used, regardless of the degree of heterogeneity. The risk of publication bias was tested using Egger's test when there were at least ten studies [19]. Visual inspection of potential publication bias through visual inspection of funnel plots exposed some studies as outliers which are defined as studies that lie outside the corresponding traditional funnel plots. The OR and 95% CI for the overall random-effects summary values were shown in forest plots.

#### Subgroup analysis

To further investigate the potential differential influence of the number of ACEs on diabetes, subgroup analyses were conducted by types of diabetes, study designs, and WHO regions including the region of the Americas (AMR), Eastern Mediterranean Region (EMR), European Region (EUR), and Western Pacific Region (WPR). Additionally, subgroup analyses were performed with adjustment of confounders including sex, age, race, education, employment, economic status, marital status, and body mass index (BMI).

#### Sensitivity analysis

Regarding the association between the number of ACEs and diabetes, sensitivity analyses were conducted to measure the overall impact of individual studies by omitting one study at a time and estimating the impact of the remaining studies (≥3 studies) to further investigate the robustness of the results.

All statistical analyses were performed using STATA version 16.0 (Stata Corporation, College Station, TX, USA). Statistical significance was considered to be *P* < 0.05 and all *P* values were two-tailed.

## RESULTS

### Search results

**Figure 1:**
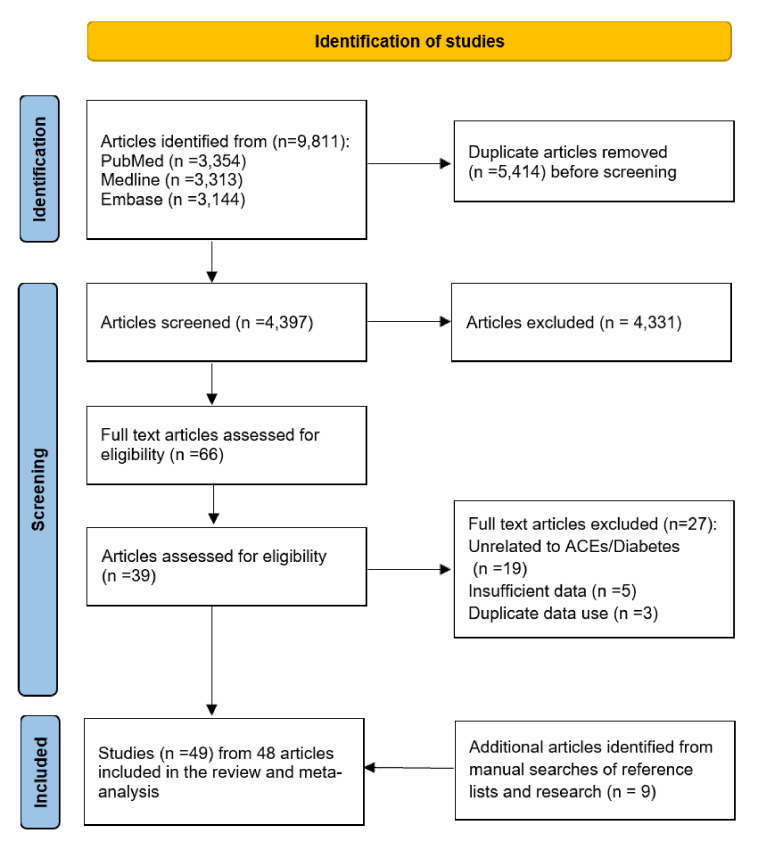
Study selection flowchart.

The literature search yielded 9811 records (PubMed N = 3354, Medline N = 3313, and Embase N = 3144). After deduplication and screening of titles and abstracts, 66 articles remained for full-text examination. An additional nine articles were identified from a manual search of reference lists. This systematic review and meta-analysis ultimately included 49 studies from 48 articles that met the inclusion and exclusion criteria, with one article reporting two studies from Japan and Finland [24]. **Figure 1** shows the entire process of study selection (PRISMA flowchart).

### Characteristics of the included studies

The 49 included studies involved 3 279 271 participants for quantitative synthesis. One study by Felitti et al. [20] was published in 1998 and republished in 2019, so only the latest version was included. Included studies were published between 2000 and 2022 and conducted in 21 countries. There were 30 cross-sectional studies and 19 cohort studies. The number of participants ranged from 89 to 2 153 164. Eight studies focused on females, one focused on males, and 40 examined mixed-sex samples. Both the number and types of ACEs were reported in 14 studies, while 19 studies only reported the number of ACEs and 16 studies only presented types of ACEs. The characteristics of included studies are presented in Table S2 in the **Online Supplementary Document**.

All eligible studies received a score of six or above in the quality assessment, with a mean score of 7.16 for cohort studies and 7.57 for cross-sectional studies, demonstrating at least moderate methodological quality. The quality assessment of included studies is presented in Table S3 and S4 in the **Online Supplementary Document**.

### Statistical results

#### Meta-analysis for the number of ACEs and diabetes

The association between the number of ACEs and diabetes is shown in **Table 1**. According to the continuous ACEs, participants exposed to one or more ACEs were at a significantly higher risk of having diabetes (per each additional ACE: OR = 1.06, 95% CI = 1.02-1.10). Those who were exposed to any ACE had a greater risk of diabetes (OR = 1.22, 95% CI = 1.16-1.28) than those who were not. Because various studies applied different reference groups, we adopted experiencing no ACEs or experiencing less than 4 ACEs as the reference category, respectively. With those exposed to no ACEs as the reference group, participants exposed to 4 or more ACEs had a higher risk of diabetes (OR = 1.44, 95% CI = 1.27-1.63). However, there was no significant association between ≥4 ACEs and diabetes (OR = 1.10, 95% CI = 0.85-1.42), when compared to those exposed to <4 ACEs. The *P*-values from Egger’s test were all higher than 0.10. As for the combined funnel plots and results of Egger’s test, there was no obvious publication bias in the number of ACEs and risk of diabetes. More details about the forest plots, funnel plots, and Egger’s test are shown in Figures S1-S14 in the **Online Supplementary Document**.

**Table 1:** Random-effects model for the number of ACEs and risk of diabetes

Number of ACEs	Reference	Studies	OR (95% CI)	*I^2^*	*Q* test
Continuous ACEs	-	9	1.06 (1.02-1.10)	68.1%	0.002
Any ACE	0 ACEs	26	1.22 (1.16-1.28)	77.3%	<0.001
1 ACE	0 ACEs	21	1.08 (1.04-1.12)	0.0%	0.734
2 ACEs	0 ACEs	16	1.24 (1.14-1.35)	37.1%	0.068
3 ACEs	0 ACEs	15	1.33 (1.20-1.46)	34.7%	0.091
≥4 ACEs	0 ACEs	17	1.44 (1.27-1.63)	77.5%	<0.001
≥4 ACEs	<4 ACEs	3	1.10 (0.85-1.42)	30.4%	0.238

ACEs – adverse childhood experiences, OR – odds ratio, CI – confidence interval

#### Meta-analysis for types of ACEs and diabetes

The pooled results of the associations between 12 types of ACEs and diabetes are shown in **Table 2**. For 12 types of ACEs, we did the meta-analysis with not having experienced the corresponding type of ACE as the reference. For two types of ACEs (ie, a family member with substance abuse and family member with mental disorder), we also did meta-analysis with not having experienced any ACE as the reference. When not having experienced the corresponding type of ACE was used as the reference, this study found statistically significant associations between the following and diabetes: economic adversity (OR = 1.11, 95% CI = 1.04-1.19), physical abuse (OR = 1.14, 95% CI = 1.07-1.21), sexual abuse (OR = 1.25, 95% CI = 1.12-1.39), verbal abuse (OR = 1.11, 95% CI = 1.03-1.20), and incarceration (OR = 1.22, 95% CI = 1.03-1.45). However, with this reference, there was no statistically significant association between neglect (OR = 1.27, 95% CI = 0.95-1.70) and the risk of diabetes. Emotional abuse (OR = 0.95, 95% CI = 0.78-1.15), domestic violence (OR = 1.08, 95% CI = 0.95-1.22), parental divorce or separation (OR = 1.11, 95% CI = 0.99-1.24), parental death (OR = 1.08, 95% CI = 0.81-1.46), family member with substance abuse (OR = 1.07, 95% CI = 0.96-1.20) or mental disorder (OR = 1.04, 95% CI = 0.96-1.12) yielded similar results. There was no strong association between having a family member with substance abuse (OR = 0.91, 95% CI = 0.78-1.05) or mental disorder (OR = 0.71, 95% CI = 0.37-1.36) and the risk of diabetes when not having experienced any ACE was used as the reference. More details about forest plots are shown in Figures S15-S28 in the **Online Supplementary Document**.

**Table 2:** Random-effects model for types of ACEs and risk of diabetes

Type of ACEs	Reference	Studies	OR (95% CI)	*I^2^*	*Q* test
Economic adversity	None	7	1.11 (1.04-1.19)	20.2%	0.276
Neglect	None	6	1.27 (0.95-1.70)	75.4%	0.001
Emotional abuse	None	3	0.95 (0.78-1.15)	0.0%	0.906
Physical abuse	None	16	1.14 (1.07-1.21)	29.8%	0.125
Sexual abuse	None	16	1.25 (1.12-1.39)	71.3%	<0.001
Verbal abuse	None	5	1.11 (1.03-1.20)	0.0%	0.772
Domestic violence	None	8	1.08 (0.95-1.22)	41.2%	0.104
Parental divorce/separation	None	8	1.11 (0.99-1.24)	27.8%	0.206
Parental death	None	3	1.08 (0.81-1.46)	76.4%	0.015
Incarceration	None	6	1.22 (1.03-1.45)	36.9%	0.161
Family member with substance abuse	None	11	1.07 (0.96-1.20)	57.9%	0.008
	No ACEs	3	0.91 (0.78-1.05)	28.9%	0.245
Family member with mental disorder	None	9	1.04 (0.96-1.12)	0.0%	0.743
	No ACEs	2	0.71 (0.37-1.36)	92.1%	<0.001

ACEs – adverse childhood experiences, OR – odds ratio, CI – confidence interval, none – not having experienced the corresponding type of ACE, no ACEs – not having experienced any ACE

Additionally, the associations between some specific types of ACEs and diabetes were not able to be assessed via meta-analysis due to a limited number of eligible studies. However, the results in those individual studies provided some insight into the potential associations between these types of ACEs and the risk of diabetes. For example, Lown et al. [25] mentioned that the association between non-traditional living situations (eg, not living with two biological parents, living with grandparents or foster parents, or living in an orphanage or group home) and T2DM was not significant (OR = 1.13, 95% CI = 0.93-1.36). Friedman et al. [26] also found that academic, interpersonal, or legal issues and the death/illness of a loved one were not associated with diabetes. However, Scott et al. [27] found other loss of parents like adoption, and foster care was associated with diabetes (OR = 1.58, 95% CI = 1.12-2.23), while Bengtsson et al. [28] mentioned that foster care (OR = 1.10, 95% CI = 0.95-1.27) and parental long-term unemployment (OR = 1.00, 95% CI = 0.95-1.05) were not associated with T1DM. Furthermore, Amemiya et al. [24] found that fear of a family member was significantly associated with diabetes in Japan (OR = 1.42, 95% CI *=* 1.15-1.76), but not in Finland (OR = 1.12, 95% CI = 0.95-1.33). Subramaniam et al. [29] found that the association between bullying and diabetes was not statistically significant (OR = 2.90, 95% CI = 0.70-12.8), whereas Thomas et al. [30] found humiliation was not associated with T2DM (OR = 1.15, 95% CI = 0.74-1.80). Alastalo et al. [31] mentioned that war evacuees and separation from parents were associated with T2DM (OR = 1.40, 95% CI = 1.10-1.90). Lastly, Zhang et al. [32] found that loneliness was associated with diabetes (OR = 1.44, 95% CI = 1.09-1.90), but that unsafe neighbourhood (OR = 1.11, 95% CI = 0.85-1.22), poor family relations (OR = 1.01, 95% CI = 0.81-1.27), and poor self-rated health in childhood (OR = 0.94, 95% CI = 0.73-1.34) were not associated with diabetes.

#### Subgroup analysis for the number of ACEs and diabetes

Subgroup analysis for the number of ACEs and diabetes was conducted based on types of diabetes, study designs, WHO regions, and adjustment of confounders including sex, age, race, education, employment, economic status, marital status, and BMI in ACE groups. There were seven ACE-groups: continuous ACEs, any ACE vs 0 ACEs, 1 ACE vs 0 ACEs, 2 ACEs vs 0 ACEs, 3 ACEs vs 0 ACEs, ≥4 ACEs vs 0 ACEs, ≥4 ACEs vs <4 ACEs. When sub-grouped by types of outcomes, positively significant associations with diabetes were found in all seven ACE groups. Similar associations were also found in cross-sectional studies, studies with adjustment of sex, age, or marital status. When sub-grouped by adjustment for BMI, positively significant associations with non-adjustment for BMI were found in all ACE groups except one (≥4 ACEs vs <4 ACEs). When sub-grouped by WHO regions, positively significant associations in studies from AMR and EMR were found in all ACE groups except two (continuous ACEs, ≥4 ACEs vs <4 ACEs). Similar associations were also found in studies with adjustments for race or economic status, and non-adjustment for employment status. When sub-grouped by adjustment for race, positively significant associations with non-adjustment for the race in all but three ACE groups (2 ACEs vs 0 ACEs, ≥4 ACEs vs 0 ACEs, ≥4 ACEs vs <4 ACEs). When sub-grouped by adjustment for economic status, positively significant associations with non-adjustment for economic status were found in all but two ACE groups (2 ACEs vs 0 ACEs, ≥4 ACEs vs <4 ACEs). When sub-grouped by study design or adjustment for marital status, studies with cohort design and non-adjustment for marital status showed a positive association in three groups (any ACE vs 0 ACEs, 3 ACEs vs 0 ACEs, ≥4 ACEs vs 0 ACEs). When sub-grouped by types of outcomes, positively significant associations with non-GDM were found in two groups (any ACE vs 0 ACEs, ≥4 ACEs vs 0 ACEs). A similar association was also found in studies conducted on WPR. When sub-grouped by types of outcomes, positively significant associations with T2DM were found in all ACE groups except three groups (1 ACE vs 0 ACEs, 3 ACEs vs 0 ACEs, ≥4 ACEs vs <4 ACEs). When sub-grouped by adjustment for education, positively significant associations in studies with non-adjustment for education were found in all but three ACE groups (1 ACE vs 0 ACEs, 2 ACEs vs 0 ACEs, ≥4 ACEs vs <4 ACEs). When sub-grouped by adjustment for employment, positively significant associations in studies with adjustment for employment were found in all ACE groups except one (3 ACEs vs 0 ACEs). Studies conducted on EUR showed positive associations between exposures and outcomes in two ACE groups (continuous ACEs, any ACE vs 0 ACEs). More detailed results regarding subgroup analysis are presented in Table S5 in the **Online Supplementary Document**.

#### Sensitivity analysis for the number of ACEs and diabetes

A sensitivity analysis was performed to assess the robustness of the found association between the number of ACEs and diabetes. The overall impact of individual studies was measured by omitting one study at a time and estimating the impact of the remaining studies. The sensitivity results did not show meaningful changes to the main results. More detailed results regarding sensitivity analysis are presented in Table S6 in the **Online Supplementary Document**.

## DISCUSSION

### Summary of the main results

Overall, ACEs were found to be associated with a higher risk of diabetes in adulthood. This study found that the more ACEs one had experienced, the more likely one was to develop diabetes. Anyone who had any type of ACE had a higher risk of developing diabetes compared with individuals who never experienced any ACE. Regarding different types of ACEs, physical abuse, verbal abuse, sexual abuse, childhood economic adversity, and incarceration were all associated with an increased risk of diabetes later in life.

### The number of ACEs and risk of diabetes

This systematic review and meta-analysis shows that cumulative ACEs have a significantly negative effect on metabolic outcomes, which corresponds with results from another meta-analysis by Jakubowski et al. [33]. There is evidence that the accumulation of disadvantages is more detrimental to children's health than any isolated specific disadvantage [34]. However, the underlying mechanisms between ACEs and diabetes remain unclear. Based on current evidence, the association between ACEs and diabetes may occur via biological, psychological, and social pathways. Biological theories, such as Herzman's theory of biological embedding, posit that early adversity alters the development of stress and inflammatory reaction through the hypothalamic-pituitary-adrenal (HPA) axis [35], influencing an individual's metabolic and inflammatory response to stress with time [36]. The dysregulated HPA axis secretes higher levels of glucocorticoids and catecholamines among chronically stressed individuals, causing insulin resistance and inflammation, which affect glucose metabolism [35]. All these changes have the potential to cause diabetes [37,38]. In terms of psychological pathways, ACEs are associated with mental illness [39]. For example, people who suffer from ACEs are vulnerable to depression [40,41], which is a risk factor for diabetes [42]. People with ACEs are also more likely to have posttraumatic stress disorder [43], which is strongly associated with the development of obesity and diabetes [44,45]. Behaviours play an important role in social pathways: people who have a greater number of ACEs are more likely to get involved in health risk behaviours like alcohol use and smoking, which can make them more prone to obesity [8]. There is evidence that ACEs are associated with inactivity and a high BMI [7]. Furthermore, people with ACEs may choose health-damaging behaviours as a coping strategy in the face of much negative stress due to abnormalities in physiological development [46]. These harmful behaviours may increase the risk of diabetes [47].

### Specific types of ACEs and risk of diabetes

In studies that considered the cumulative impact of ACEs, each type of ACE is assumed as having equal health risks. However, not all types of ACEs are equal and different types of ACEs may have different pathways and levels of impact on diabetes [48]. Furthermore, ACEs can have intergenerational impacts due to genetic or behavioural factors [49].

Early economic adversity is significantly associated with diabetes, as shown by Kyrou et al. [50]. People who have experienced economic adversity tend to be in unfavourable circumstances and engage in health risk behaviors like consuming calorie-dense foods, lacking physical activity [51], and smoking more frequently, behaviours which are associated with diabetes [52]. Adults who suffered from hunger in childhood will tend to become obese due to a loss of internal balance in hunger-satiety regulation and a tendency to increase food intake without hunger [53]. Psychologically they will feel self-abased, stressed, and vulnerable [54] and such conditions can be perpetuated across generations with adverse health effects [54].

The association between abuse and diabetes is concerning. Abuse not only leads to health risk behaviours, but also causes persistent increases in inflammatory markers like interleukin-6 (IL-6) [55], tumor necrosis factor-α (TNF-α) [56], and C-reactive protein (CRP) [57], which may add to the risk of inflammation-related diseases like diabetes and alter the pathogenic mechanisms of gene expression [58,59]. Moreover, physical abuse reduces a people's sense of control, impairs family relationships, and leads to poor mental and physical health [60]. Sexual abuse leads women to be more likely to experience considerably psychological symptomatology, and have frequent suicide attempts, substance addiction, and undergo battery [61]. Diabetes is also associated with the incarceration of a family member during childhood. Children of incarcerated parents are aware of society's stigma regarding criminal behaviours, feel shame, and may take part in harmful behaviours, as well as experience cognitive delays, developmental degradation, and inappropriate coping strategies, resulting in long-term psychological effects or immediate neurophysiological changes [62]. One study revealed a complex association between witnessing incarceration-related events and HPA axis function, suggesting that witnessing a father's arrest is a stressful or even traumatic experience for a child, that glucocorticoid responses are blunted, and that there is heterogeneity in the response compared to those who experience relatively normative stress [63].

However, several types of ACEs were not found to be significantly associated with diabetes. For example, this contrasts the meta-analysis by Huang et al. [9], which found a strong association between neglect and T2DM. The inconsistencies between studies may be due to the differences in the number of included studies and the type of diabetes of interest. An additional consideration is that the impact of these types of ACEs is oftentimes obscured by other types of more influential ACEs. Adversity rarely occurs in isolation and therefore it is common for several ACEs to appear together and exert combined effects on the development of diabetes. For example, a meta-analysis focusing on the effect of multiple ACEs on health reported that 57% of 252 467 participants across all studies had at least one ACE and 13% of 244 979 participants had at least four ACEs [7]. To fully understand the association between types of ACEs and diabetes, more studies are needed in the future.

In the subgroup analysis, varying numbers of ACEs were associated with diabetes, but such evidence regarding different types of diabetes remained inconsistent. For example, the associations between ACEs and non-GDM or T2DM were not clear, but statistically non-significant for GDM. The primary reason is that most included did not clearly report the type of diabetes or adopted a mixed type of diabetes, making the evidence regarding a specific type of diabetes largely lacking. The etiology of different types of diabetes is different, so it is necessary that future studies figure out associations between ACEs and different types of diabetes. The results of adjusting for sex, age, and marital status in the model demonstrate that disparities in these covariates can influence the association between ACEs and the development of diabetes. That means the effect of sex, age, and marital status in ACEs on diabetes is complex, and more sex-specific, age-specific, and marital status-specific studies are needed in the future. When the model was adjusted for BMI, the association between ACEs and diabetes was not positively significant, which aligns with the previously mentioned association between ACEs and BMI, as BMI is an important factor for diabetes.

### Strengths and limitation

This review and meta-analysis has several strengths. First, it is the first review and meta-analysis to use both the number and types of ACEs as exposures and different types of diabetes as the outcome. Second, the study involved a rigorous literature search with a detailed data extraction process, ensuring the use of high-quality data. Third, a sufficient sample size allowed for a comprehensive analysis of a wide range of exposures and outcomes. Fourth, subgroup and sensitivity analyses both support the robustness of the associations between cumulative ACEs and diabetes. Fifth, by using self-reported ACEs, the study is more likely to reflect the actual severity of ACEs influence, especially abuse, which is often hidden, unknown, or not fully reported by officials [64-66].

Despite these strengths, our study also remains limited due to several shortcomings. First, there is heterogeneity among studies since they use different questionnaires to measure ACEs, including self-reported questionnaires that are prone to recall bias. Second, there is a lack of studies on peer relationships, such as bullying, and this may have affected the results regarding these ACEs. Third, ACEs tend to occur simultaneously and are highly correlated. Examining one type of ACE instead of multiple ACEs may lead to an overestimation of the effect of a single exposure. Future studies should investigate the interaction between different types of ACEs.

## CONCLUSIONS

ACEs may have a cumulative effect on the risk of diabetes in adulthood. Understanding the association between ACEs and diabetes might aid in the development of strategies to prevent diabetes. There is a need to raise awareness about the prevalence and effects of ACEs and to establish effective prevention and early intervention strategies for ACEs. It is also crucial to improve the capacity to help people with ACEs strengthen resilience and form healthy lifestyles. Furthermore, more studies are needed to better understand the underlying mechanisms of the association between ACEs and different types of diabetes, so that we can develop better strategies to combat the consequences of ACEs and simultaneously reduce the disease burden of diabetes.

## Additional material:


Online Supplementary Document
