# Supplementary material for: Adverse childhood experiences and risk of diabetes: A systematic review and meta-analysis
Source: J Glob Health. 2022 Nov 2;12:04082. doi: 10.7189/jogh.12.04082 (PMC9624439; doi:10.7189/jogh.12.04082)
Supplement: Online Supplementary Document [file jogh-12-04082-s001.pdf]

ONLINE SUPPLEMENTARY DOCUMENT

Title: Adverse childhood experiences and risk of diabetes: a systematic review and meta-analysis

Authors: Siyu Zhu, Shiyi Shan, Wen Liu, Shuting Li, Leying Hou, Xuanyin Huang, Yi Liu, Qian Yi, Weidi Sun, Kun Tang, Davies Adeloye, Igor Rudan, Peige Song; Global Health Epidemiology Research Group (GHERG)

Table S1. Search strategy

Table S2. Characteristics of included studies (n=49)

Table S3. Quality assessment of cross-sectional studies (n=30)

Table S4. Quality assessment of cohort studies (n=19)

Table S5. Subgroup meta-analysis for the number of ACEs and risk of diabetes

Table S6. Sensitivity meta-analysis for the number of ACEs and risk of diabetes

Figure S1. Forest plot for 1 ACE vs 0 ACEs and risk of diabetes

Figure S2. Forest plot for 2 ACEs vs 0 ACEs and risk of diabetes

Figure S3. Forest plot for 3 ACEs vs 0 ACEs and risk of diabetes

Figure S4. Forest plot for ≥4 ACEs vs 0 ACEs and risk of diabetes

Figure S5. Forest plot for continuous ACEs and risk of diabetes

Figure S6. Forest plot for any ACE vs 0 ACEs and risk of diabetes

Figure S7. Forest plot for ≥4 ACEs vs <4 ACEs and risk of diabetes

Figure S8. Funnel plot to detect publication bias for 1 ACE vs 0 ACEs and risk of diabetes, Egger test, P=0.293

Figure S9. Funnel plot to detect publication bias for 2 ACEs vs 0 ACEs and risk of diabetes, Egger test, P=0.378

Figure S10. Funnel plot to detect publication bias for 3 ACEs vs 0 ACEs and risk of diabetes, Egger test, P=0.289

Figure S11. Funnel plot to detect publication bias for ≥4 ACEs vs 0 ACEs and risk of diabetes, Egger test, P=0.669

Figure S12. Funnel plot to detect publication bias for continuous ACEs and risk of diabetes

Figure S13. Funnel plot to detect publication bias for any ACE vs 0 ACEs and risk of diabetes, Egger test, P=0.362

Figure S14. Funnel plot to detect publication bias for ≥4 ACEs vs <4 ACEs and risk of diabetes

Figure S15. Forest plot for economic adversity and risk of diabetes, Reference: none

Figure S16. Forest plot for neglect and risk of diabetes, Reference: none

Figure S17. Forest plot for emotional abuse and risk of diabetes, Reference: none

Figure S18. Forest plot for physical abuse and risk of diabetes, Reference: none

Figure S19. Forest plot for sexual abuse and risk of diabetes, Reference: none

Figure S20. Forest plot for verbal abuse and risk of diabetes, Reference: none

Figure S21. Forest plot for domestic violence and risk of diabetes, Reference: none

Figure S22. Forest plot for parental divorce/separation and risk of diabetes, Reference: none

Figure S23. Forest plot for parental death and risk of diabetes, Reference: none

Figure S24. Forest plot for incarceration and risk of diabetes, Reference: none

Figure S25. Forest plot for family member with substance abuse and risk of diabetes, Reference: none

Figure S26. Forest plot for family member with substance abuse and risk of diabetes, Reference: no ACEs

Figure S27. Forest plot for family member with mental disorder and risk of diabetes, Reference: none

Figure S28. Forest plot for family member with mental disorder and risk of diabetes, Reference: no ACEs

Table S1. Search strategy

Database	Search terms		Results
**PubMed**	#1	(("ACE"[Title/Abstract] OR "ACEs"[Title/Abstract] OR "adverse childhood experience*"[Title/Abstract] OR "adverse childhood event*"[Title/Abstract] OR "childhood adversit*"[Title/Abstract]) AND "diabet*"[Title/Abstract] AND 2000/01/01:3000/12/31[Date - Publication]) AND (humans[Filter])	3,354
**Medline**	#1	exp Diabetes Mellitus/	475,965
	#2	Diabet*.ab,ti.	708,214
	#3	1 or 2	772,492
	#4	(ACE or ACEs or adverse childhood experience* or adverse childhood event* or childhood adversity*).ab,ti.	42,171
	#5	3 and 4	5,249
	#6	limit 5 to humans	4,332
	#7	limit 6 to yr="2000 -Current"	3,383
	#8	limit 7 to journal article	3,313
**Embase**	#8	#7 AND ([article]/lim OR [article in press]/lim)	3,144
	#7	#6 AND [2000-2021]/py	7,349
	#6	#5 AND [humans]/lim	8,484
	#5	#3 AND #4	9,957
	#4	'ACE':ab,ti or 'ACEs':ab,ti or 'adverse childhood experience*':ab,ti or 'adverse childhood event*':ab,ti or 'childhood adversit*':ab,ti	63,408
	#3	#1 or #2	1,326,704
	#2	'Diabet*':ab,ti	1,084,826
	#1	'Diabetes Mellitus'/exp	1,139,377

Table S2. Characteristics of included studies (n=49)

Authors (year)	Country	Study design	Number of participants	Sex	Age	Exposure	Outcome assessment	Outcome	Number of ACEs	Type of ACEs
Goodwin, et al. (2004)(1)	US	Cross-sectional	5,877	Male and Female	Range:15-54	Physical abuse, sexual abuse, neglect	Self-reported diabetes diagnosis (based on a checklist of physical illnesses)	Diabetes	No	Yes
Thomas, et al. (2008)(2)	UK	Cohort	9,310	Male and Female	At 45 years	Emotional neglect, physical neglect, household dysfunction, abuse	Self-reported T2DM diagnosis	T2DM	No	Yes
Alastalo, et al. (2009)(3)	Finland	Cohort	2,003	Male and Female	Mean±SD:63.7±2.8(war evacuees), 61.1±2.8(non-separated controls)	War evacuees, separated	Use of medication for chronic, physician-diagnosed diseases (diabetes), a 2-hour 75-g oral glucose tolerance	T2DM	No	Yes
Ramiro, et al. (2010)(4)	Philippines	Cross-sectional	1,068	Male and Female	Range:35-92	Childhood abuse (psychological/emotional abuse, physical abuse, sexual abuse, physical neglect, psychological neglect), household dysfunction (illicit drug use, alcohol abuse, mental illness, mother treated	Self-rated health	Diabetes	Yes	No

Authors (year)	Country	Study design	Number of participants	Sex	Age	Exposure	Outcome assessment	Outcome	Number of ACEs	Type of ACEs
						violently, incarcerated household member, parental separation or divorce)				
Rich-Edwards, et al. (2010)(5)	US	Cohort	67,853	Female	Baseline: at 25–42 years in 1989, follow up to 2005	Physical abuse, sexual abuse	Self-reported T2DM diagnosis	T2DM	No	Yes
Anda, et al. (2010)(6)	US	Cross-sectional	7,471	Male and Female	Range:18+	Physical abuse, sexual abuse, verbal abuse, household mental illness, household substance abuse, parental divorce/separation, witnessed domestic violence, incarcerated household member	Self-reported diabetes diagnosis	Non-GDM	Yes	Yes
Scott, et al. (2011)(7)	Mexico,US, Belgium,France,Germany,Italy,Netherlands,Spain,Japan,Colombia	Cross-sectional	18,303	Male and Female	Range:18+	Physical abuse, sexual abuse, neglect, parental death, parental divorce, other parental loss, parental mental disorder, parental substance use, parental criminal behavior, family violence, family economic adversity	Self-reported diabetes diagnosis	Diabetes	Yes	Yes
Widom, et al. (2012)(8)	US	Cohort	1,575	Male and Female	Mean:41.2	Physical abuse, sexual abuse, neglect	Physical examination	Diabetes	No	Yes
Lynch, et al.	US	Cross-section	801	Male and	Range:19-82	Abuse, neglect, household	Any T2DM diagnosis	T2DM	Yes	No

Authors (year)	Country	Study design	Number of participants	Sex	Age	Exposure	Outcome assessment	Outcome	Number of ACEs	Type of ACEs
(2013)(9)		al		Female		dysfunction	(based on clinical chart diagnoses)			
Ye, et al. (2014)(10)	US	Cross-section al	5,928	Male and Female	Range:18+	Family dysfunction (family member with mental illness, family member with substance abuse, family member in prison, family separation or divorce, witnessing domestic violence), abuse (physical abuse, verbal abuse, sexual abuse)	Self-reported diabetes diagnosis	Diabetes	Yes	Yes
McCauley, et al. (2015)(11)	US	Cross-section al	36,485	Female	Mean±SE:50.5±1.14(Veteran), 49.4±0.18(Non-veteran)	Household dysfunction (parental mental illness, incarceration, domestic violence, divorce, alcohol abuse, and illicit substance use), childhood abuse (emotional abuse, physical abuse, touched sexually, forced to touch someone else sexually, or forced to have sex)	Diagnosis by a health care professional of diabetes	Diabetes	Yes	No
Gilbert, et al. (2015)(12)	US	Cross-section al	53,998	Male and Female	Range:18+	Physical abuse, sexual abuse, emotional abuse, and household member mental illness, alcoholism, drug abuse,	Self-reported non-gestational diabetes diagnosis	Non-GDM	Yes	No

Authors (year)	Country	Study design	Number of participants	Sex	Age	Exposure	Outcome assessment	Outcome	Number of ACEs	Type of ACEs
						imprisonment, divorce, intimate partner violence				
McCrory, et al. (2015)(13)	Ireland	Cross-section al	6,912	Male and Female	Range:50+	Disadvantaged socioeconomic circumstances, parental substance abuse, physical abuse, sexual abuse	Self-reported diabetes diagnosis	Diabetes	Yes	Yes
Bellis, et al. (2015)(14)	UK	Cross-section al	3,885	Male and Female	Range:18-69	Physical abuse, verbal abuse, sexual abuse, parental separation, exposure to domestic violence and growing up in a household with mental illness, alcohol abuse, drug abuse or incarceration	Self-reported T2DM diagnosis	T2DM	Yes	No
Friedman, et al. (2015)(15)	US	Cross-section al	3,996	Male and Female	Range:25-74	Academic, interpersonal, physical/sexual abuse, work or financial problems, death or illness of a loved one, legal problems, and parental substance abuse	Self-reported diabetes diagnosis	Diabetes	Yes	Yes

Authors (year)	Country	Study design	Number of participants	Sex	Age	Exposure	Outcome assessment	Outcome	Number of ACEs	Type of ACEs
Duncan, et al. (2015)(16)	US	Cohort	14,493	Male and Female	Range:24-34	Sexual abuse, physical abuse, emotional abuse, neglect	Physical examination and/or self-report of previous diabetes diagnosis by a doctor	Diabetes	No	Yes
Monnat, et al. (2015)(17)	US	Cross-section al	52,250	Male and Female	Range:18-64	Physical abuse, sexual abuse, verbal abuse, witnessing parental domestic violence, experiencing parental divorce, living with anyone who was depressed, mentally ill or suicidal, living with anyone who was a problem drinker or alcoholic, living with anyone who abused drugs, living with anyone who was incarcerated	Self-reported diabetes diagnosis	Diabetes	No	Yes
Almuneef, et al. (2016)(18)	Saudi Arabia	Cross-section al	10,156	Male and Female	Range:18-88	Family dysfunction, physical, sexual and emotional abuse and neglect by parents or caregivers,	Received a medical diagnosis of diabetes	Diabetes	Yes	No

Authors (year)	Country	Study design	Number of participants	Sex	Age	Exposure	Outcome assessment	Outcome	Number of ACEs	Type of ACEs
						peer violence, witnessing community violence, and exposure to collective violence				
Campbell, et al. (2016)(19)	US	Cross-sectional	48,526	Male and Female	Range:18+	Physical abuse, sexual abuse, verbal abuse, parental substance abuse, lived with mentally ill, domestic violence	Self-reported diabetes diagnosis	Diabetes	Yes	Yes
Wade Jr, et al. (2016)(20)	US	Cross-sectional	1,784	Male and Female	Range:18-97	Conventional ACE (physical abuse, sexual abuse, emotional abuse, emotional neglect, physical neglect, substance abuse in the home, mentally ill household member, domestic violence, and household member in prison), Expanded ACE (experiencing racism, witnessing violence, bullying, experiencing foster care, and living in unsafe neighborhoods)	Self-reported diabetes diagnosis	Diabetes	Yes	Yes
Mason, et al. (2016)(21)	US	Cohort	45,550	Female	Baseline: at 25–42 years in 1989, follow up to 2001 and 2009	Physical abuse, sexual abuse	Any GDM diagnosis (based on medical record)	GDM	No	Yes

Authors (year)	Country	Study design	Number of participants	Sex	Age	Exposure	Outcome assessment	Outcome	Number of ACEs	Type of ACEs
Ford, et al. (2016)(22)	UK	Cross-sectional	5,621	Male and Female	Range:18-69	Sexual abuse, physical abuse, verbal abuse, parental separation, mental illness, alcohol abuse, drug abuse, incarceration	Not reported	T2DM	Yes	No
Shields, et al. (2016)(23)	Canada	Cross-sectional	21,878	Male and Female	Range:18+	Physical abuse, sexual abuse, intimate partner violence	Self-reported diabetes diagnosis	Diabetes	No	Yes
Wade, et al. (2017)(24)	US	Cross-sectional	71,413	Male and Female	Range:18+	Household stressors (parental separation/divorce, household alcoholism, household mental illness, domestic violence in the home, household illicit or prescription drug use, incarcerated household member), abuse (emotionally abused, physically abused, touched sexually, forced to touch sexually, forced to have sex)	Self-reported diabetes diagnosis	Diabetes	Yes	Yes
Llabre, et al. (2017)(25)	US	Cohort	5,117	Male and Female	Range:18-74	Emotional abuse, physical abuse, sexual abuse, emotional neglect, physical neglect, parental separation or divorce, witnessing female parent being	Physical examination and/or self-report of previous diabetes diagnosis by a doctor	Diabetes	Yes	No

Authors (year)	Country	Study design	Number of participants	Sex	Age	Exposure	Outcome assessment	Outcome	Number of ACEs	Type of ACEs
						abused, living with a substance abuser, living with a mentally ill person, imprisonment of a household member				
Chanlongbutra, et al. (2018)(26)	US	Cross-sectional	79,810	Male and Female	Range:18+	Household dysfunction (parental mental illness, incarceration, domestic violence, divorce, alcohol abuse, illicit substance use), childhood abuse (emotional abuse, physical abuse, touched sexually, forced to touch someone else sexually, or forced to have sex)	Self-reported health care professional diagnosis	Diabetes	Yes	No
Lown, et al. (2019)(27)	US	Cohort	8,377	Male and Female	Range:14-56	Childhood poverty, parental death, adverse living situation, living with a mentally ill person, living with a problem drinker, being physically abused as a child	Self-reported T2DM diagnosis	T2DM	Yes	Yes
Amemiya, et al. (2019)(28)	Japan	Cross-sectional	13,123	Male and Female	Range:65-74	Parental divorce, witness of domestic violence or physical abuse, financial difficulties in the family	Self-reported diabetes diagnosis	Diabetes	Yes	Yes

Authors (year)	Country	Study design	Number of participants	Sex	Age	Exposure	Outcome assessment	Outcome	Number of ACEs	Type of ACEs
Amemiya, et al. (2019)(28)	Finland	Cohort	10,353	Male and Female	Mean±SD:64.4±2.9	Parental divorce, fear of a family member, financial difficulties in the family	Any diabetes diagnosis (based on records in national health registers)	Diabetes	Yes	Yes
Carrillo-Vega, et al. (2019)(29)	Mexico	Cross-sectional	8,848	Male and Female	Range:50-80	No shoes during childhood, went to bed hungry before 10 years	Self-reported diabetes diagnosis	Diabetes	No	Yes
Felitti, et al. (2019)(30)	US	Cohort	9,508	Male and Female	Range:19+	Psychological abuse, physical abuse, sexual abuse, violence against mother, or living with household members who were substance abusers, mentally ill or suicidal, or ever imprisoned	Self-reported diabetes diagnosis	Diabetes	Yes	No
Merrick, et al. (2019)(31)	US	Cross-sectional	144,017	Male and Female	Range:18+	Physical abuse, emotional abuse, sexual abuse, household member substance misuse, incarceration, mental illness, parental divorce, or witnessing intimate partner violence	Self-reported diabetes diagnosis	Diabetes	Yes	No

Authors (year)	Country	Study design	Number of participants	Sex	Age	Exposure	Outcome assessment	Outcome	Number of ACEs	Type of ACEs
Kreatsoulas, et al. (2019)(32)	US	Cross-sectional	45,482	Male and Female	Range:18-99	Neglect (depression in home, alcoholic in home, illicit drug use in home, parent/ guardian in prison), violence/ emotional abuse (physical abuse between parents, physical abuse, emotional abuse), sexual abuse (been sexually touched, forced to sexually touch them, sexual intercourse)	Self-reported diabetes diagnosis	Diabetes	Yes	No
Salas, et al. (2019)(33)	US	Cross-sectional	78,435	Male and Female	Range:18+	Household dysfunction (living with anyone who was depressed/mentally ill/suicidal, a problem drinker/alcoholic, a drug user/abuser, or incarcerated, having separated or divorced parents, or living in home where adult abuse was witnessed), physical abuse, verbal abuse, and sexual abuse (touched sexually, forced to	Self-reported non-gestational diabetes diagnosis	Non-GDM	No	Yes

Authors (year)	Country	Study design	Number of participants	Sex	Age	Exposure	Outcome assessment	Outcome	Number of ACEs	Type of ACEs
						touch someone else, or forced to have sex) prior to 18 years of age				
Schoenaker, et al. (2019)(34)	Australia	Cohort	6,317	Female	Baseline: at 18–23 years in 1996, follow up to 2015	Physical abuse, emotional abuse, sexual abuse, household dysfunction (parental substance abuse, parental separation or divorce, mental illness, mother treated violently, criminal behavior)	Self-reported GDM diagnosis	GDM	Yes	Yes
White, et al. (2020)(35)	US	Cross-sectional	23,045	Male and Female	Range:40+	Physical abuse, sexual abuse, verbal abuse, parental substance abuse, lived with mentally ill, domestic violence	Self-reported non-gestational diabetes diagnosis	Non-GDM	No	Yes

Authors (year)	Country	Study design	Number of participants	Sex	Age	Exposure	Outcome assessment	Outcome	Number of ACEs	Type of ACEs
El Mhamdi, et al. (2020)(36)	Tunisia	Cross-section al	2,120	Male and Female	Range:18+	Intra-familial early life adversities experienced in the home (conflictual relationship with parents/caregivers, neglect, household dysfunction, physical abuse, sexual abuse), Social early life adversities experienced in the society (peer violence, witnessing community violence, exposure to collective violence)	Self-reported diabetes diagnosis	Diabetes	Yes	No
Ittoop, et al. (2020)(37)	US	Cross-section al	89	Male and Female	Range:18+	Physical abuse, verbal abuse, sexual abuse, physical neglect, emotional neglect, a parent who's an alcoholic, a mother who's a victim of domestic	Any non-gestational diabetes diagnosis (based on records in medical charts)	Non-GDM	Yes	No

Authors (year)	Country	Study design	Number of participants	Sex	Age	Exposure	Outcome assessment	Outcome	Number of ACEs	Type of ACEs
						violence, a family member in jail, a family member diagnosed with a mental illness, and the disappearance of a parent through divorce, death or abandonment				
Stanhope, et al. (2020)(38)	US	Cohort	2,319	Female	Range:18-74	Abuse (physical, sexual, and emotional), neglect (physical and emotional), parental separation, witnessing maternal abuse, living with a substance abuser, mentally ill person in the household, and household member imprisonment	Self-reported GDM diagnosis	GDM	Yes	No
Upadhyaya, et al. (2020)(39)	Finland	Cohort	754	Male	Range:42-60	Parental alcohol problems, parental divorce	Any T2DM diagnosis (based on records in Care Register for Health Care (CRHC))	T2DM	No	Yes

Authors (year)	Country	Study design	Number of participants	Sex	Age	Exposure	Outcome assessment	Outcome	Number of ACEs	Type of ACEs
Flores-Torres, et al. (2020)(40)	Mexico	Cohort	9,853	Female	Range:25+	Abuse (physical, sexual, and emotional), neglect (physical and emotional), household challenges (household exposure to parental separation or divorce, physical violence, substance abuse, mental illness, household member incarceration)	Self-reported diagnosis, treatment, and diabetes-related complications	Diabetes	Yes	Yes
Bengtsson, et al. (2020)(41)	Denmark	Cohort	2,153,164	Male and Female	Birth between 1 January 1980 and 31 December 2015	Family dynamics (i.e., being placed in foster care, parental or sibling psychiatric illness, parental alcohol or drug abuse, and parental separation), loss or threat of loss within the family	Any T1DM diagnosis (based on records in nationwide registers)	T1DM	No	Yes

Authors (year)	Country	Study design	Number of participants	Sex	Age	Exposure	Outcome assessment	Outcome	Number of ACEs	Type of ACEs
						(i.e., death of a parent or a sibling and parental or sibling somatic illness) and social disadvantage (i.e., family poverty and parental long-term unemployment)				
Waehrer, et al. (2020)(42)	US	Cross-section al	132,551	Male and Female	Range:18+	Parent divorce, incarceration, substance use, depression, domestic violence, child physical abuse, verbal abuse, sexual abuse	Self-reported diabetes diagnosis	Diabetes	Yes	No
Bala, et al. (2020)(43)	US	Cohort	3,184	Female	Range:14+	Have an adult who can be trusted to help, live with parents or guardians who got divorced or separated, live with incarcerated parents or guardians, live with parents or guardians with substance abuse,	Self-reported diabetes diagnosis	Diabetes	Yes	No

Authors (year)	Country	Study design	Number of participants	Sex	Age	Exposure	Outcome assessment	Outcome	Number of ACEs	Type of ACEs
						move because of problems paying the rent or mortgage, food deprivation, in foster care				
Subramaniam, et al. (2021)(44)	Singapore	Cross-sectional	6,126	Male and Female	Range:18+	Physical abuse, sexual abuse, emotional abuse and neglect by parents or caregivers, family dysfunction	Self-reported diabetes diagnosis	Diabetes	Yes	Yes
Almuneef, et al. (2021)(45)	Saudi Arabia	Cross-sectional	10,156	Male and Female	Range:18+	Sexual abuse	Self-reported diabetes diagnosis	Diabetes	No	Yes
Versteegen, et al. (2021)(46)	US	Cohort	300	Female	Range:18-40	Psychological abuse, physical abuse, sexual abuse, violence against mother, or living with household members who were substance abusers, mentally ill or suicidal, or ever imprisoned	Any GDM diagnosis (based on medical record)	GDM	Yes	No
Lin, et al. (2021)(47)	China	Cohort	11,972	Male and Female	Range:45+	Physical abuse, emotional neglect, household substance abuse, household mental illness, domestic violence, incarcerated	Self-report diagnosis or a physician's diagnosis or in combination with	Diabetes	Yes	No

Authors (year)	Country	Study design	Number of participants	Sex	Age	Exposure	Outcome assessment	Outcome	Number of ACEs	Type of ACEs
						household member, parental separation or divorce, unsafe neighborhood and bullying, parental death, sibling death, parental disability	health assessment and medication data			
Zhang, et al. (2022)(48)	China	Cohort	17,115	Male and Female	Range:45+	Early maternal death, early paternal death, father: illiteracy, father: farming, hunger, economic hardship, loneliness, neighborhood, poor family relations, abuse from mother, abuse from father, poor self-rated health	Self-report diabetes diagnosis	Diabetes	No	Yes

Notes: ACEs - adverse childhood experiences, T1DM - type 1 diabetes mellitus, T2DM - type 2 diabetes mellitus, GDM - gestational diabetes mellitus, No - not report the number or type of ACEs, Yes - report the number or type of ACEs, US - the United States, UK - the United Kingdom.

Table S3. Quality assessment of cross-sectional studies (n=30)

Authors (year)	Define the source of information	List of inclusion and exclusion criteria	Indicate time period	Subjects consecutive	Evaluators of subjective components	Quality assurance	Exclusion from analysis	Confounding control	Missing data	Completeness of data	Completeness of follow-up	Total score
Goodwin, et al. (2004)(1)	1	1	1	1	1	0	0	1	0	1	0	7
Ramiro, et al. (2010)(4)	1	0	1	1	1	0	0	1	0	1	0	6
Anda, et al. (2010)(6)	1	0	1	1	0	0	0	1	1	1	0	6
Scott, et al. (2011)(7)	1	1	1	1	1	0	1	1	0	1	0	8
Lynch, et al. (2013)(9)	1	1	1	1	1	1	1	1	0	0	0	8
Ye, et al. (2014)(10)	1	1	1	1	1	0	0	1	0	1	0	7
McCauley, et al. (2015)(11)	1	1	1	1	1	1	1	1	0	1	0	9
Gilbert, et al. (2015)(12)	1	1	1	1	1	0	0	1	0	1	0	7

Authors (year)	Define the source of information	List of inclusion and exclusion criteria	Indicate time period	Subjects consecutive	Evaluators of subjective components	Quality assurance	Exclusion from analysis	Confounding control	Missing data	Completeness of data	Completeness of follow-up	Total score
McCrory, et al. (2015)(13)	1	1	0	1	1	0	0	1	1	1	0	7
Bellis, et al. (2015)(14)	1	1	1	1	1	0	0	1	0	1	0	7
Friedman, et al. (2015)(15)	1	1	1	1	1	0	1	1	1	1	0	9
Monnat, et al. (2015)(17)	1	1	1	1	0	0	1	1	1	1	0	8
Almuneef, et al. (2016)(18)	1	0	1	1	1	1	0	1	1	0	0	7
Campbell, et al. (2016)(19)	1	1	1	1	1	0	0	1	0	1	0	7
Wade Jr, et al. (2016)(20)	1	1	1	1	1	0	0	1	0	1	0	7
Ford, et al. (2016)(22)	1	1	1	1	1	0	1	1	0	1	0	8

Authors (year)	Define the source of information	List of inclusion and exclusion criteria	Indicate time period	Subjects consecutive	Evaluators of subjective components	Quality assurance	Exclusion from analysis	Confounding control	Missing data	Completeness of data	Completeness of follow-up	Total score
Shields, et al. (2016)(23)	1	1	1	1	0	0	1	1	1	1	0	8
Wade, et al. (2017)(24)	1	0	1	1	1	0	0	1	1	1	0	7
Chanlongbutra, et al. (2018)(26)	1	1	1	1	1	0	0	1	0	0	0	6
Amemiya, et al. (2019)(28)	1	1	1	1	1	0	1	1	1	1	0	9
Carrillo-Vega, et al. (2019)(29)	1	1	1	1	1	0	1	1	1	1	0	9
Merrick, et al. (2019)(31)	1	1	1	1	1	0	1	1	0	1	0	8
Kreatsoulas, et al. (2019)(32)	1	1	1	1	1	0	1	1	1	0	0	8

Authors (year)	Define the source of information	List of inclusion and exclusion criteria	Indicate time period	Subjects consecutive	Evaluators of subjective components	Quality assurance	Exclusion from analysis	Confounding control	Missing data	Completeness of data	Completeness of follow-up	Total score
Salas, et al. (2019)(33)	1	1	1	1	1	0	0	1	1	1	0	8
White, et al. (2020)(35)	1	1	1	1	1	0	1	1	1	1	0	9
El Mhamdi, et al. (2020)(36)	1	1	1	1	1	0	0	1	0	0	0	6
Ittoop, et al. (2020)(37)	1	1	1	1	1	1	0	1	1	0	0	8
Waehrer, et al. (2020)(42)	1	1	1	1	1	0	0	1	1	0	0	7
Subramaniam, et al. (2021)(44)	1	1	1	1	1	0	0	1	0	1	0	7
Almuneef, et al. (2021)(45)	1	1	1	1	1	0	1	1	1	1	0	9

Table S4. Quality assessment of cohort studies (n=19)

Authors (year)	Selection				Comparability of cohorts	Outcome			
	Representativeness of the exposed cohort	Selection of the nonexposed cohort	Ascertainment of exposure	Outcome of interest	Comparability	Assessment of outcome	Folllow-up duration	Adequacy of follow up of cohorts	Total scores
Thomas, et al. (2008)(2)	1	1	0	1	2	0	1	1	7
Alastalo, et al. (2009)(3)	1	1	0	1	2	1	1	1	8
Rich-Edwards, et al. (2010)(5)	0	1	0	1	2	0	1	1	6
Widom, et al. (2012)(8)	1	1	0	1	2	1	1	0	7
Duncan, et al. (2015)(16)	1	1	0	1	2	1	1	1	8
Mason, et al. (2016)(21)	0	1	0	1	2	1	1	0	6
Llabre, et al. (2017)(25)	1	1	0	1	2	0	1	1	7
Lown, et al. (2019)(27)	1	1	0	1	2	0	1	1	7
Amemiya, et al. (2019)(28)	0	1	0	1	2	1	1	1	7
Felitti, et al. (2019)(30)	1	1	0	1	2	0	1	1	7
Schoenaker, et	1	1	0	1	2	0	1	1	7

Authors (year)	Selection				Comparability of cohorts	Outcome			Total scores
	Representativeness of the exposed cohort	Selection of the nonexposed cohort	Ascertainment of exposure	Outcome of interest	Comparability	Assessment of outcome	Folllow-up duration	Adequacy of follow up of cohorts	
al. (2019)(34)									
Stanhope, et al. (2020)(38)	1	1	0	1	2	0	1	1	7
Upadhyaya, et al. (2020)(39)	1	1	0	1	2	1	1	1	8
Flores-Torres, et al. (2020)(40)	0	1	0	1	2	0	1	1	6
Bengtsson, et al. (2020)(41)	1	1	0	1	2	1	1	1	8
Bala, et al. (2020)(43)	1	1	0	1	2	1	1	0	7
Versteegen, et al. (2021)(46)	1	1	0	1	2	1	1	1	8
Lin, et al. (2021)(47)	1	1	0	1	2	1	1	1	8
Zhang, et al. (2022)(48)	1	1	0	1	2	1	1	0	7

Table S5. Subgroup meta-analysis for number of ACEs and risk of diabetes

	Continuous ACEs		Any ACE vs 0 ACEs		1 ACE vs 0 ACEs		2 ACEs vs 0 ACEs		3 ACEs vs 0 ACEs		≥4 ACEs vs 0 ACEs		≥4 ACEs vs <4 ACEs	
	**Studies**	**OR (*95% CI*)**	**Studies**	**OR (*95% CI*)**	**Studies**	**OR (*95% CI*)**	**Studies**	**OR (*95% CI*)**	**Studies**	**OR (*95% CI*)**	**Studies**	**OR (*95% CI*)**	**Studies**	**OR (*95% CI*)**
**Type of DB**														
Diabetes	5	**1.07 (1.02, 1.12)**	20	**1.20 (1.13, 1.26)**	17	**1.08 (1.04, 1.12)**	14	**1.23 (1.12, 1.35)**	12	**1.32 (1.17, 1.48)**	11	**1.36 (1.17, 1.58)**	1	**1.20 (1.06, 1.36)**
Non-GDM	1	1.23 (0.89, 1.72)	2	**1.29 (1.18, 1.41)**	1	1.30 (0.99, 1.70)	1	1.31 (0.94, 1.82)	1	1.47 (0.98, 2.20)	2	**1.40 (1.20, 1.65)**	-	-
T2DM	1	**1.11 (1.01, 1.22)**	3	**1.33 (1.19, 1.49)**	3	1.10 (0.93, 1.31)	1	**1.31 (1.02, 1.69)**	1	1.27 (0.89, 1.82)	3	**2.03 (1.20, 3.44)**	-	-
GDM	2	0.97 (0.90, 1.04)	1	1.12 (0.95, 1.32)	-	-	-		1	1.29 (0.92, 1.81)	1	1.49 (1.00, 2.22)	2	0.88 (0.58, 1.32)
**Study design**													-	-
Cohort	4	1.02 (0.93, 1.12)	7	**1.17 (1.10, 1.25)**	3	1.05 (0.92, 1.20)	3	1.16 (0.98, 1.37)	5	**1.21 (1.04, 1.41)**	5	**1.47 (1.18, 1.82)**	2	0.88 (0.58, 1.32)
Cross-sectional	5	**1.08 (1.06, 1.10)**	19	**1.23 (1.16, 1.31)**	18	**1.08 (1.04, 1.13)**	13	**1.26 (1.15, 1.39)**	10	**1.39 (1.24, 1.55)**	12	**1.42 (1.22, 1.66)**	1	**1.20 (1.06, 1.36)**
**WHO region**														
AMR	7	1.04 (1.00, 1.09)	14	**1.19 (1.15, 1.24)**	12	**1.08 (1.04, 1.12)**	10	**1.21 (1.12, 1.31)**	10	**1.36 (1.25, 1.47)**	11	**1.34 (1.23, 1.47)**	3	1.10 (0.85, 1.42)
WPR	1	1.08 (1.00, 1.17)	5	**1.11 (1.04, 1.18)**	3	1.04 (0.88, 1.23)	3	1.15 (0.97, 1.37)	3	1.08 (0.90, 1.29)	3	**1.24 (1.03, 1.48)**	-	-
EMR	-	-	2	**1.66 (1.41, 1.94)**	2	**1.28 (1.08, 1.51)**	2	**1.65 (1.39, 1.95)**	2	**1.72 (1.40, 2.10)**	1	**2.38 (2.00, 2.83)**	-	-
EUR	1	**1.17 (1.06, 1.29)**	4	**1.22 (1.05, 1.42)**	3	1.02 (0.85, 1.21)	-	-	-	-	2	1.87 (0.72, 4.83)	-	-
**Adjustment for model**														
Sex														
Yes	5	**1.08 (1.02, 1.15)**	21	**1.22 (1.16, 1.29)**	19	**1.08 (1.04, 1.12)**	14	**1.26 (1.17, 1.36)**	11	**1.32 (1.16, 1.50)**	13	**1.46 (1.28, 1.67)**	1	**1.20 (1.06, 1.36)**
No	1	1.23 (0.89, 1.72)	1	0.90 (0.69, 1.18)	1	1.00 (0.76, 1.32)	1	0.70 (0.44, 1.11)	1	1.10 (0.63, 1.90)	1	0.74 (0.49, 1.12)	-	-
Age														
Yes	6	**1.07 (1.03, 1.12)**	25	**1.22 (1.16, 1.28)**	21	**1.08 (1.04, 1.12)**	16	**1.24 (1.14, 1.35)**	14	**1.33 (1.20, 1.47)**	16	**1.44 (1.26, 1.64)**	1	**1.20 (1.06, 1.36)**
No	3	0.98 (0.91, 1.07)	1	1.12 (0.95, 1.32)	-	-	-	-	1	1.29 (0.92, 1.81)	1	1.49 (1.00, 2.22)	2	0.88 (0.58, 1.32)
Race														
Yes	5	1.04 (0.99, 1.10)	16	**1.19 (1.15, 1.24)**	15	**1.08 (1.04, 1.12)**	11	**1.22 (1.15, 1.30)**	9	**1.27 (1.13, 1.42)**	12	**1.39 (1.26, 1.53)**	2	1.04 (0.71, 1.52)
No	4	**1.11 (1.04, 1.18)**	10	**1.21 (1.06, 1.39)**	6	**1.12 (1.01, 1.25)**	5	1.19 (0.86, 1.65)	6	**1.50 (1.28, 1.75)**	5	1.43 (0.93, 2.18)	1	1.31 (0.50, 3.41)

	Continuous ACEs		Any ACE vs 0 ACEs		1 ACE vs 0 ACEs		2 ACEs vs 0 ACEs		3 ACEs vs 0 ACEs		≥4 ACEs vs 0 ACEs		≥4 ACEs vs <4 ACEs	
	**Studies**	**OR (*95% CI*)**	**Studies**	**OR (*95% CI*)**	**Studies**	**OR (*95% CI*)**	**Studies**	**OR (*95% CI*)**	**Studies**	**OR (*95% CI*)**	**Studies**	**OR (*95% CI*)**	**Studies**	**OR (*95% CI*)**
Education														
Yes	6	1.05 (1.00, 1.11)	17	**1.22 (1.14, 1.29)**	15	**1.10 (1.05, 1.14)**	13	**1.26 (1.16, 1.36)**	11	**1.30 (1.16, 1.46)**	11	**1.47 (1.25, 1.71)**	2	1.04 (0.71, 1.52)
No	3	**1.09 (1.01, 1.18)**	9	**1.21 (1.11, 1.31)**	6	1.02 (0.94, 1.11)	3	1.12 (0.74, 1.69)	4	**1.43 (1.18, 1.74)**	6	**1.39 (1.07, 1.81)**	1	1.31 (0.50, 3.41)
Employment														
Yes	2	**1.12 (1.04, 1.21)**	6	**1.19 (1.12, 1.26)**	5	**1.12 (1.04, 1.20)**	4	**1.28 (1.10, 1.49)**	3	1.14 (0.81, 1.60)	4	**1.40 (1.13, 1.75)**	1	**1.20 (1.06, 1.36)**
No	7	1.04 (0.99, 1.09)	20	**1.22 (1.15, 1.30)**	16	**1.07 (1.02, 1.11)**	12	**1.23 (1.10, 1.36)**	12	**1.36 (1.23, 1.50)**	13	**1.45 (1.24, 1.70)**	2	0.88 (0.58, 1.32)
Economic status														
Yes	2	1.02 (0.95, 1.10)	12	**1.22 (1.15, 1.30)**	11	**1.13 (1.05, 1.21)**	9	**1.26 (1.15, 1.37)**	7	**1.21 (1.02, 1.43)**	9	**1.45 (1.24, 1.69)**	-	-
No	7	**1.08 (1.02, 1.13)**	14	**1.21 (1.13, 1.30)**	10	**1.06 (1.02, 1.11)**	7	1.20 (1.00, 1.44)	8	**1.42 (1.30, 1.56)**	8	**1.41 (1.15, 1.73)**	3	1.10 (0.85, 1.42)
Marital status														
Yes	3	**1.09 (1.03, 1.15)**	11	**1.22 (1.12, 1.34)**	10	**1.12 (1.06, 1.18)**	8	**1.32 (1.20, 1.45)**	6	**1.26 (1.04, 1.53)**	7	**1.47 (1.18, 1.83)**	1	**1.20 (1.06, 1.36)**
No	6	1.04 (0.97, 1.11)	15	**1.19 (1.14, 1.25)**	11	1.05 (1.00, 1.10)	8	1.14 (0.99, 1.31)	9	**1.37 (1.25, 1.51)**	10	**1.41 (1.21, 1.64)**	2	0.88 (0.58, 1.32)
BMI														
Yes	3	0.99 (0.94, 1.03)	2	1.17 (0.96, 1.42)	1	1.10 (0.69, 1.76)	1	1.30 (0.77, 2.20)	-	-	-	-	1	1.31 (0.50, 3.41)
No	6	**1.08 (1.03, 1.12)**	24	**1.22 (1.16, 1.28)**	20	**1.08 (1.04, 1.12)**	15	**1.24 (1.14, 1.35)**	15	**1.33 (1.20, 1.46)**	17	**1.44 (1.27, 1.63)**	2	1.04 (0.71, 1.52)

Notes: ACEs - adverse childhood experiences, OR - odds ratio, CI - confidence interval, T1DM - type 1 diabetes mellitus, T2DM - type 2 diabetes mellitus, GDM - gestational diabetes mellitus, Non-GDM - diabetes excluding gestational diabetes mellitus, WHO region - the World Health Organization region, AMR - countries in the WHO Region of the Americas, EMR - countries in the WHO Eastern Mediterranean Region, EUR - countries in the WHO European Region, WPR - countries in the WHO Western Pacific Region, BMI- body mass index. If data from more than one country were included in a study, they were excluded in the subgroup analysis according to the WHO region. If the adjustment for confounders was not explicitly shown in the model, this study was excluded from the subgroup analysis.

Table S6. Sensitivity meta-analysis for the number of ACEs and risk of diabetes

	Continuous ACEs		Any ACEs vs 0 ACEs		1 ACE vs 0 ACEs		2 ACEs vs 0 ACEs		3 ACEs vs 0 ACEs		≥4 ACEs vs 0 ACEs	
**Excluded 1 study at a time**	**Studies**	**OR (*95%CI*)**	**Studies**	**OR (*95%CI*)**	**Studies**	**OR (*95%CI*)**	**Studies**	**OR (*95%CI*)**	**Studies**	**OR (*95%CI*)**	**Studies**	**OR (*95%CI*)**
Combined	9	**1.06(1.01,1.10)**	26	**1.08(1.04,1.12)**	21	**1.07(1.03,1.11)**	16	**1.20(1.09,1.31)**	15	**1.28(1.14,1.42)**	17	**1.38(1.23,1.54)**
1	8	1.06(1.00,1.12)	25	**1.08(1.04,1.11)**	20	**1.07(1.03,1.11)**	15	**1.19(1.07,1.30)**	14	**1.28(1.13,1.43)**	16	**1.36(1.21,1.51)**
2	8	1.05(1.00,1.10)	25	**1.08(1.04,1.12)**	20	**1.07(1.03,1.11)**	15	**1.21(1.10,1.32)**	14	**1.29(1.15,1.43)**	16	**1.38(1.23,1.54)**
3	8	1.05(1.00,1.09)	25	**1.08(1.04,1.12)**	20	**1.07(1.03,1.11)**	15	**1.17(1.07,1.26)**	14	**1.25(1.11,1.38)**	16	**1.30(1.18,1.42)**
4	8	**1.05(1.01,1.10)**	25	**1.08(1.04,1.11)**	20	**1.07(1.03,1.11)**	15	**1.18(1.06,1.30)**	14	**1.26(1.11,1.42)**	16	**1.40(1.23,1.57)**
5	8	**1.07(1.03,1.11)**	25	**1.08(1.04,1.12)**	20	**1.07(1.03,1.11)**	15	**1.19(1.08,1.31)**	14	**1.27(1.13,1.41)**	16	**1.38(1.22,1.55)**
6	8	1.05(1.00,1.10)	25	**1.08(1.04,1.11)**	20	**1.07(1.03,1.11)**	15	**1.20(1.08,1.31)**	14	**1.27(1.12,1.42)**	16	**1.38(1.22,1.55)**
7	8	**1.06(1.01,1.11)**	25	**1.08(1.04,1.11)**	20	**1.07(1.03,1.11)**	15	**1.18(1.07,1.30)**	14	**1.29(1.14,1.43)**	16	**1.42(1.28,1.56)**
8	8	1.05(1.00,1.10)	25	**1.08(1.04,1.11)**	20	**1.07(1.03,1.11)**	15	**1.18(1.07,1.30)**	14	**1.32(1.21,1.44)**	16	**1.40(1.24,1.56)**
9	8	**1.07(1.03,1.11)**	25	**1.08(1.04,1.11)**	20	**1.07(1.03,1.11)**	15	**1.24(1.15,1.32)**	14	**1.28(1.14,1.43)**	16	**1.36(1.21,1.51)**
10			25	**1.08(1.04,1.12)**	20	**1.07(1.03,1.11)**	15	**1.20(1.08,1.31)**	14	**1.27(1.11,1.42)**	16	**1.38(1.22,1.53)**
11			25	**1.08(1.04,1.12)**	20	**1.07(1.03,1.11)**	15	**1.20(1.08,1.31)**	14	**1.28(1.14,1.43)**	16	**1.39(1.21,1.56)**
12			25	**1.08(1.04,1.11)**	20	**1.08(1.04,1.12)**	15	**1.22(1.10,1.33)**	14	**1.27(1.12,1.41)**	16	**1.38(1.22,1.55)**
13			25	**1.08(1.04,1.12)**	20	**1.07(1.03,1.11)**	15	**1.19(1.06,1.33)**	14	**1.28(1.13,1.43)**	16	**1.38(1.22,1.54)**
14			25	**1.11(1.06,1.16)**	20	**1.07(1.03,1.11)**	15	**1.20(1.09,1.32)**	14	**1.27(1.13,1.42)**	16	**1.40(1.22,1.58)**
15			25	**1.08(1.04,1.12)**	20	**1.09(1.04,1.13)**	15	**1.19(1.07,1.31)**	14	**1.32(1.19,1.46)**	16	**1.39(1.23,1.55)**
16			25	**1.08(1.04,1.12)**	20	**1.08(1.04,1.13)**	15	**1.20(1.08,1.32)**			16	**1.38(1.22,1.54)**
17			25	**1.08(1.04,1.12)**	20	**1.07(1.03,1.11)**					16	**1.41(1.24,1.57)**
18			25	**1.08(1.04,1.11)**	20	**1.07(1.03,1.11)**						
19			25	**1.08(1.04,1.11)**	20	**1.07(1.03,1.11)**						
20			25	**1.08(1.04,1.11)**	20	**1.07(1.03,1.11)**						
21			25	**1.08(1.04,1.12)**	20	**1.08(1.04,1.12)**						
22			25	**1.08(1.04,1.11)**								

	Continuous ACEs		Any ACEs vs 0 ACEs		1 ACE vs 0 ACEs		2 ACEs vs 0 ACEs		3 ACEs vs 0 ACEs		≥4 ACEs vs 0 ACEs	
**Excluded 1 study at a time**	**Studies**	**OR (*95%CI*)**	**Studies**	**OR (*95%CI*)**	**Studies**	**OR (*95%CI*)**	**Studies**	**OR (*95%CI*)**	**Studies**	**OR (*95%CI*)**	**Studies**	**OR (*95%CI*)**
23			25	**1.08(1.04,1.11)**								
24			25	**1.08(1.04,1.11)**								
25			25	**1.08(1.04,1.12)**								
26			25	**1.08(1.04,1.12)**								

Notes: ACEs - adverse childhood experiences, OR - odds ratio, CI - confidence interval.

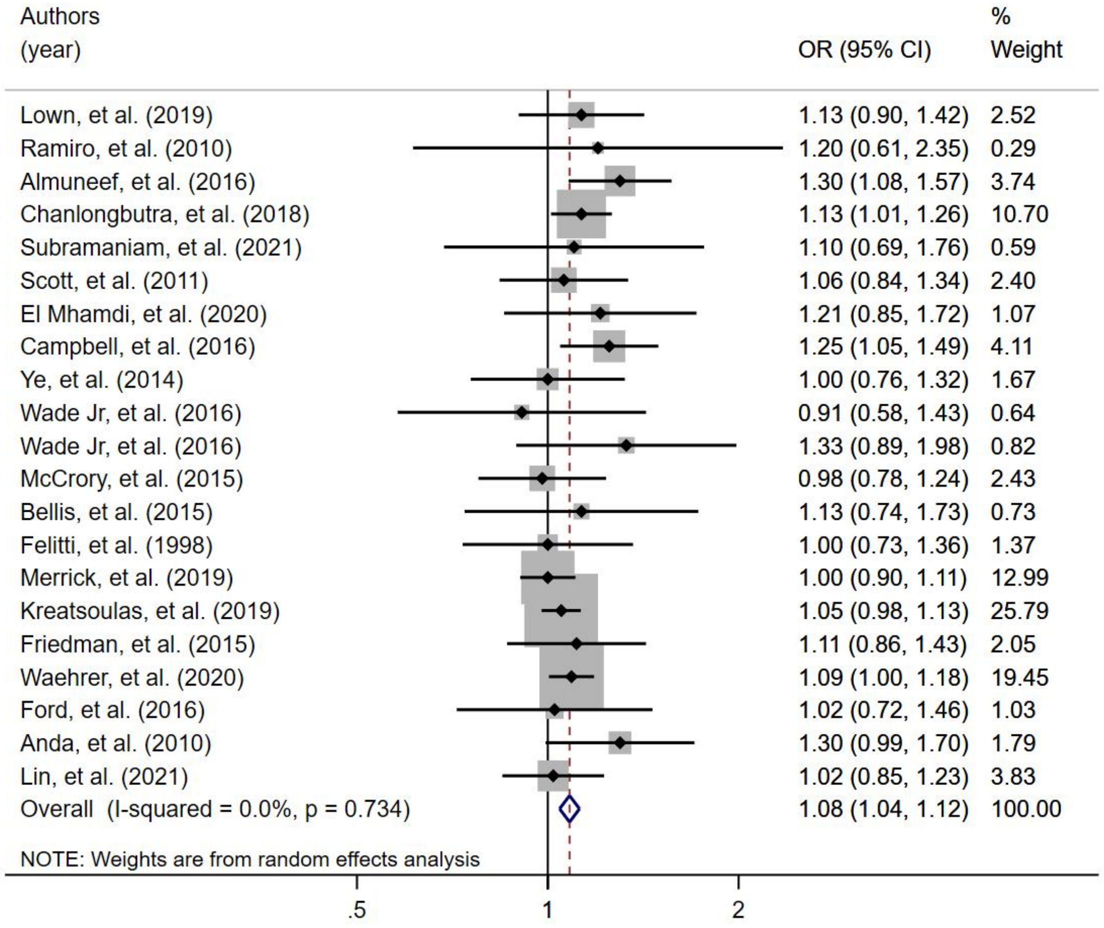

Figure S1. Forest plot for 1 ACE vs 0 ACEs and risk of diabetes

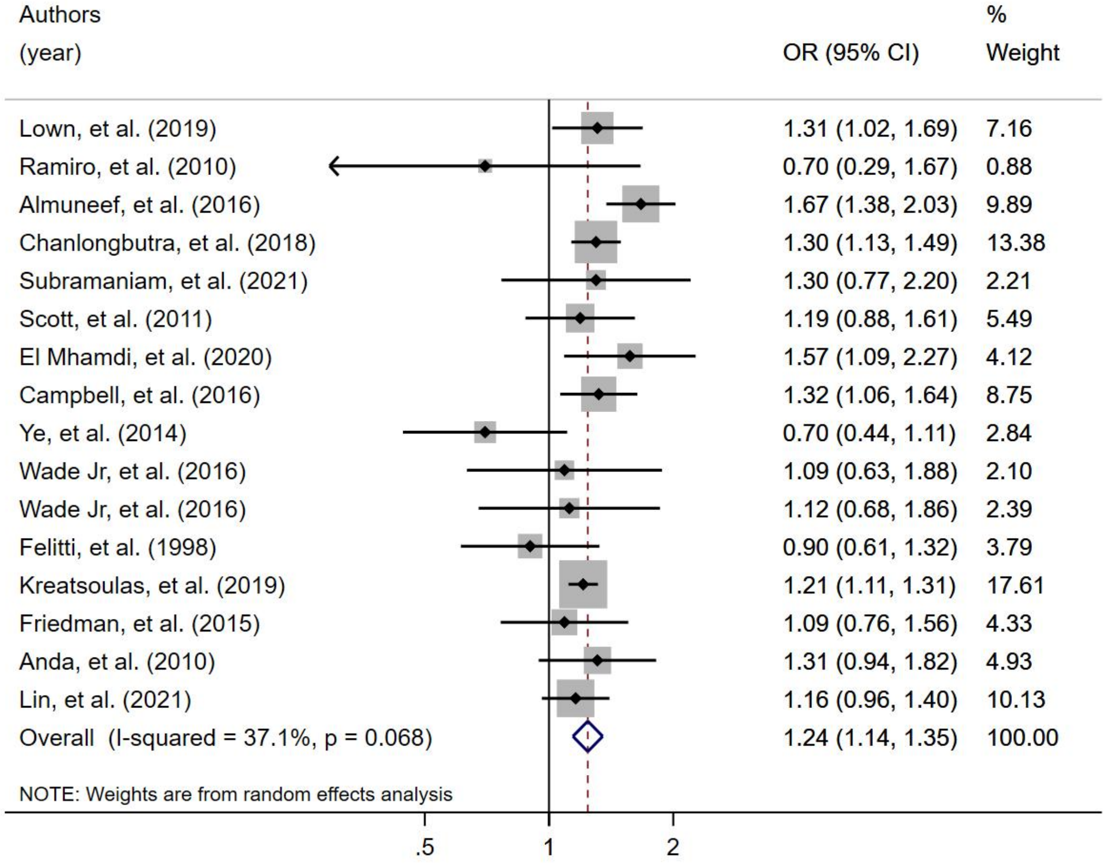

Figure S2. Forest plot for 2 ACEs vs 0 ACEs and risk of diabetes

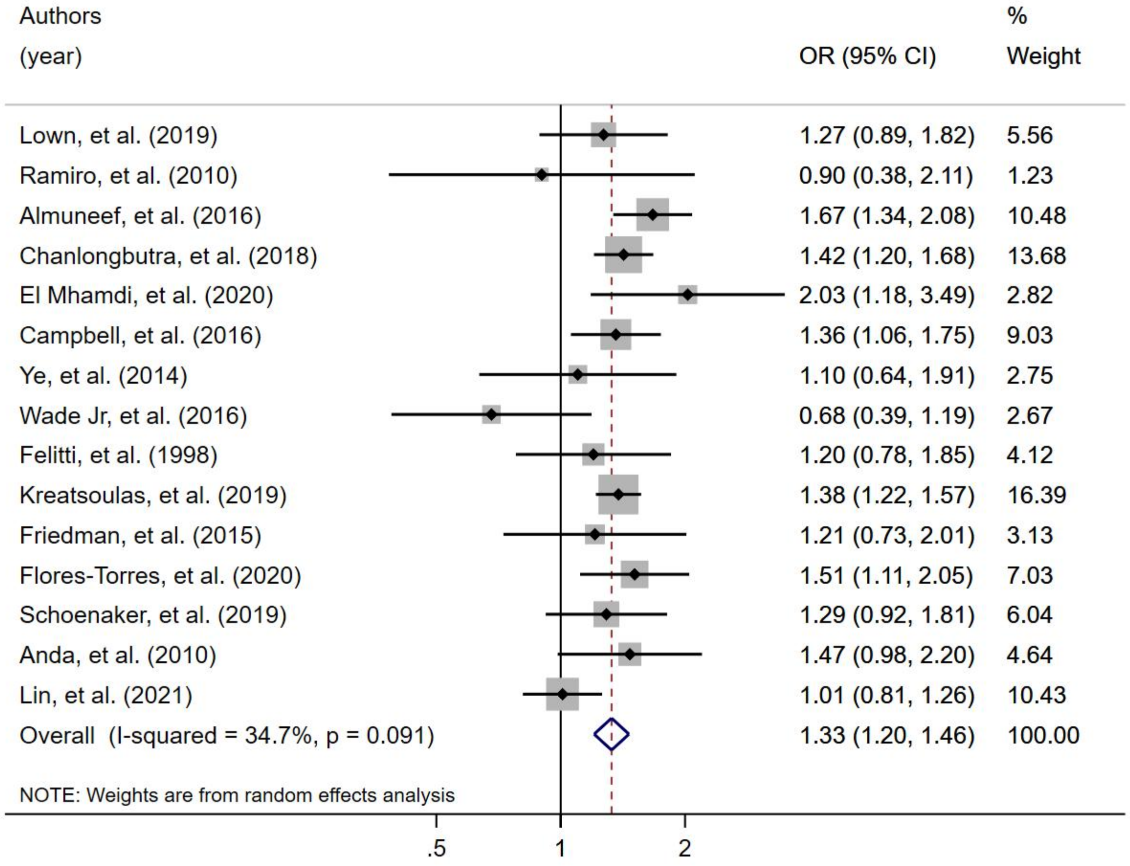

Figure S3. Forest plot for 3 ACEs vs 0 ACEs and risk of diabetes

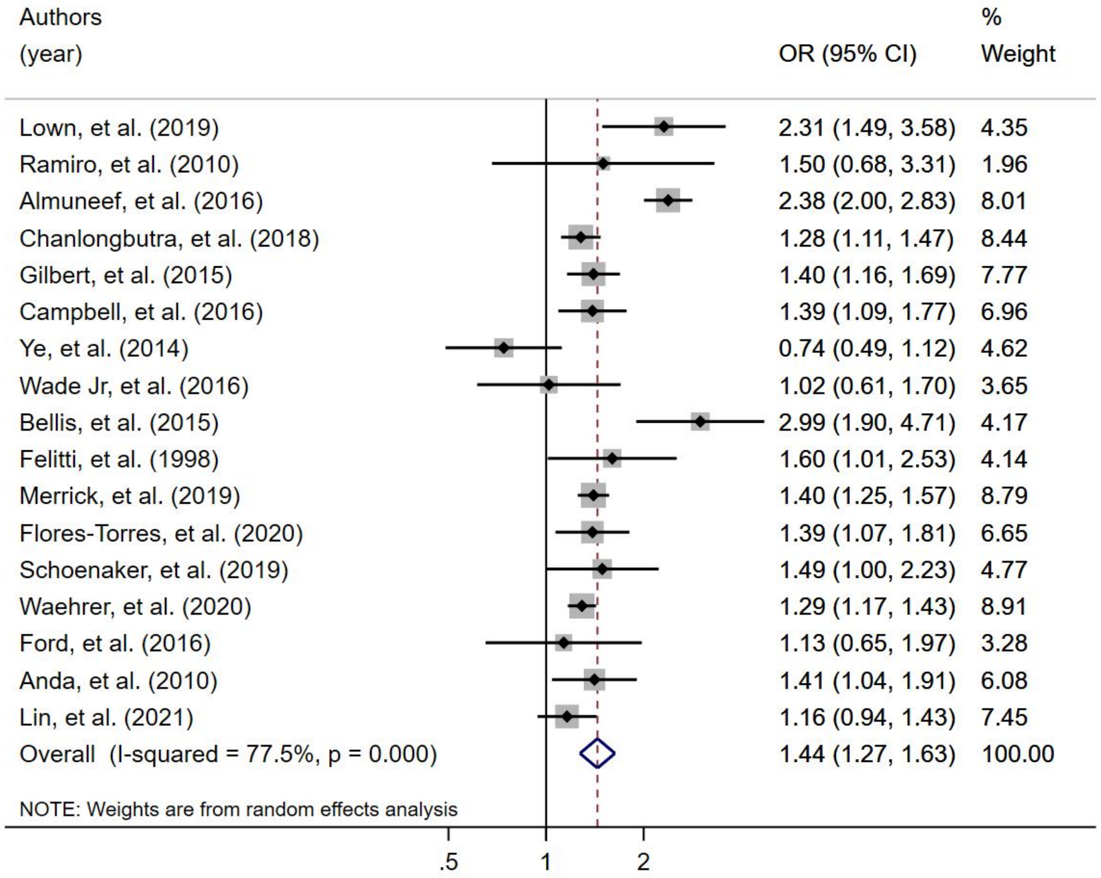

Figure S4. Forest plot for ≥4 ACEs vs 0 ACEs and risk of diabetes

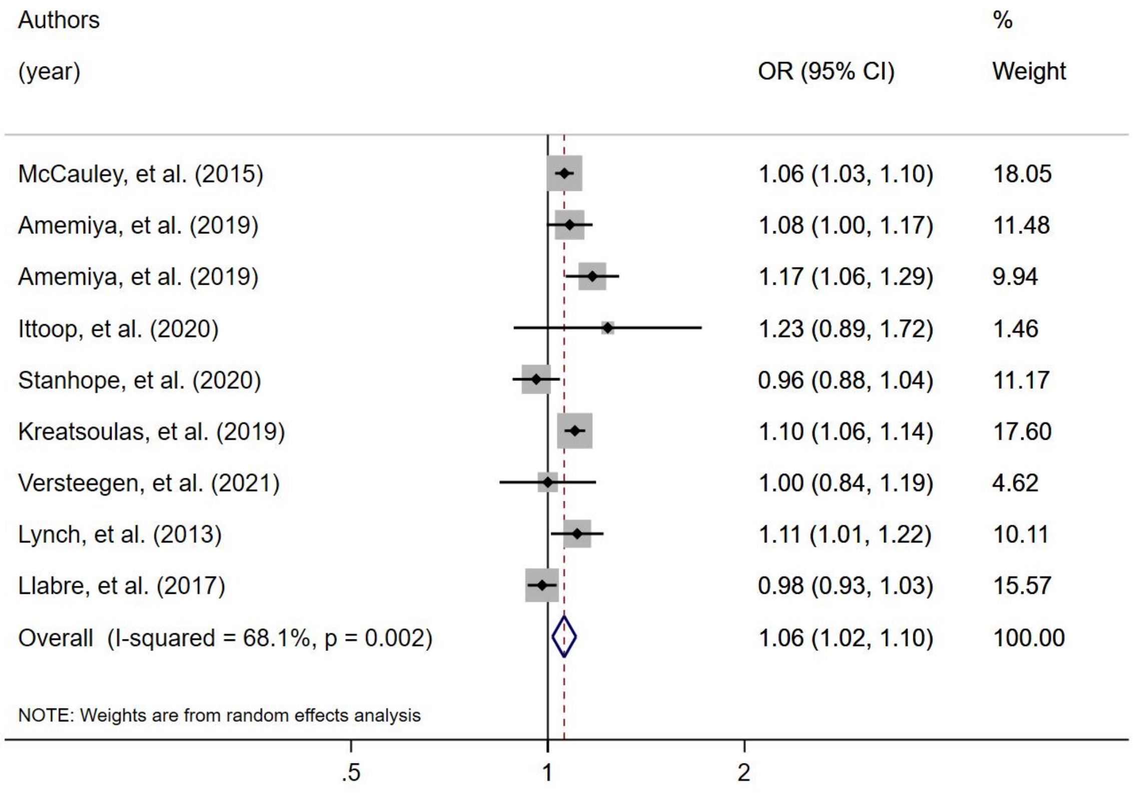

Figure S5. Forest plot for continuous ACEs and risk of diabetes

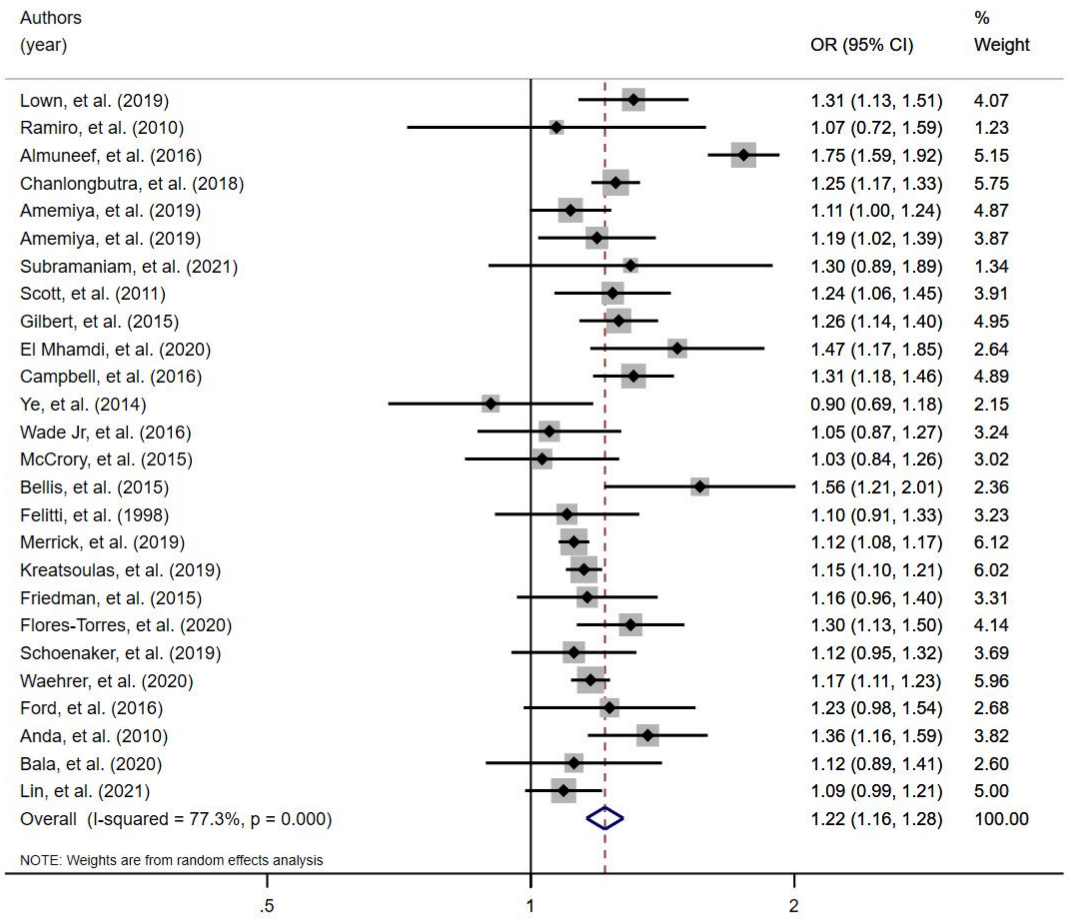

Figure S6. Forest plot for any ACE vs 0 ACEs and risk of diabetes

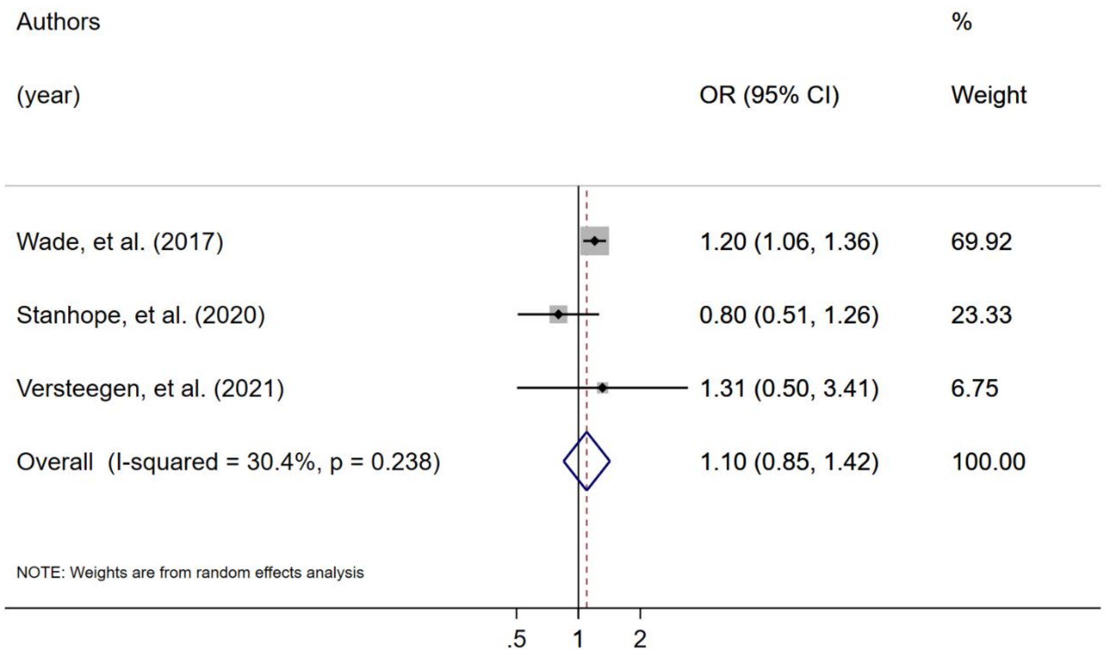

Figure S7. Forest plot for ≥4 ACEs vs <4 ACEs and risk of diabetes

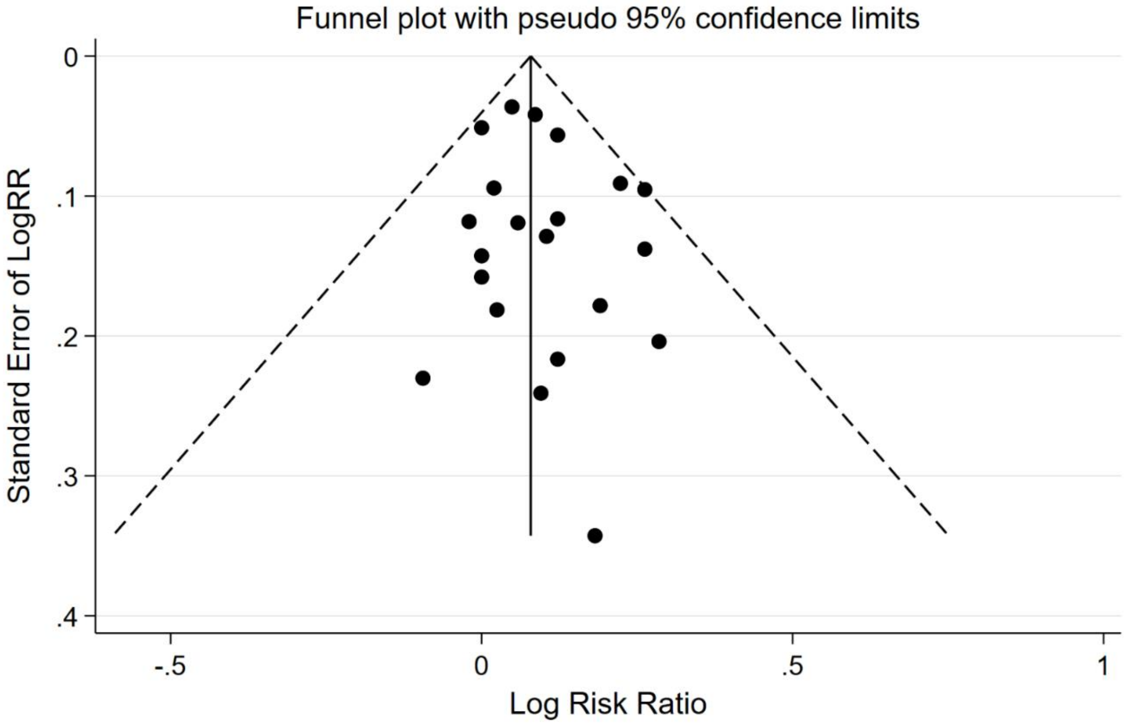

Figure S8. Funnel plot to detect publication bias for 1 ACE vs 0 ACEs and risk of diabetes, Egger test, P=0.293

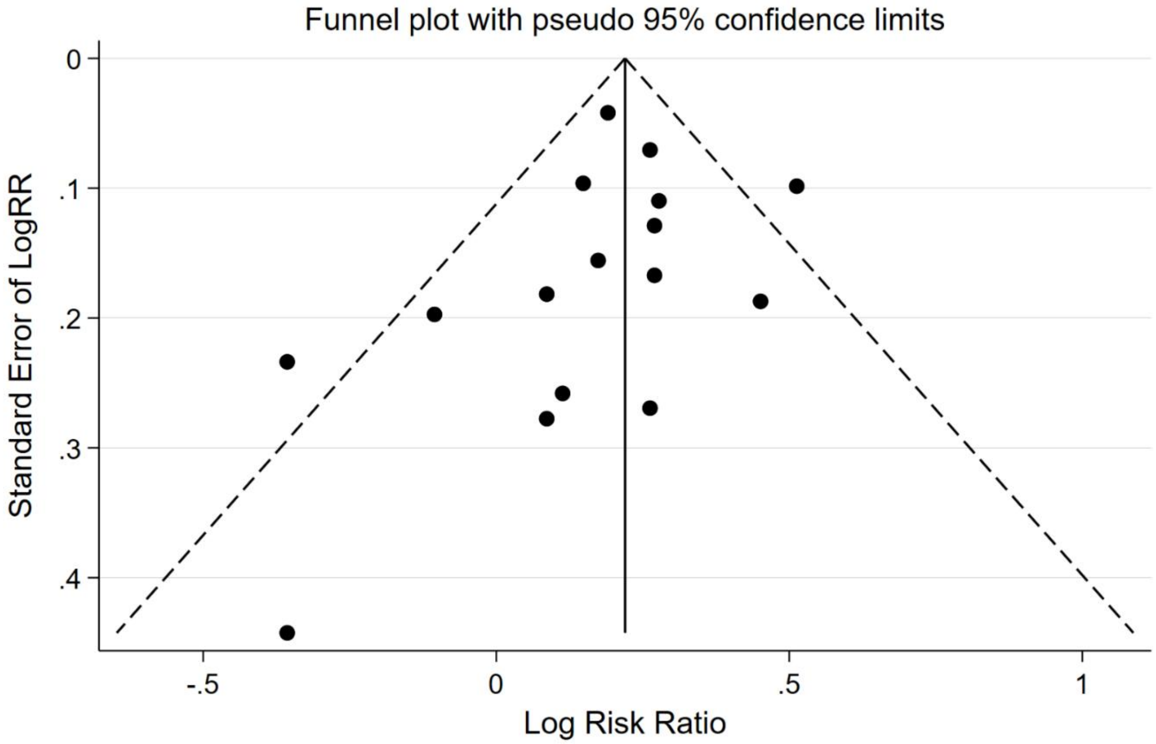

Figure S9. Funnel plot to detect publication bias for 2 ACEs vs 0 ACEs and risk of diabetes, Egger test, P=0.378

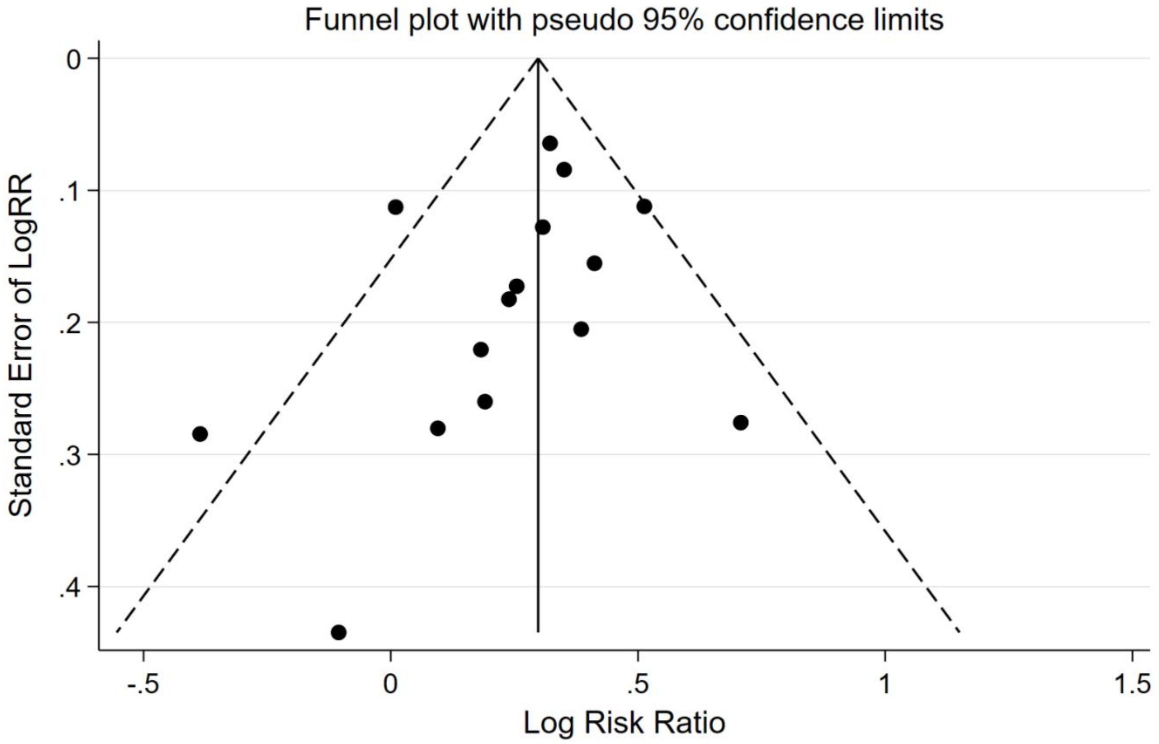

Figure S10. Funnel plot to detect publication bias for 3 ACEs vs 0 ACEs and risk of diabetes, Egger test, P=0.289

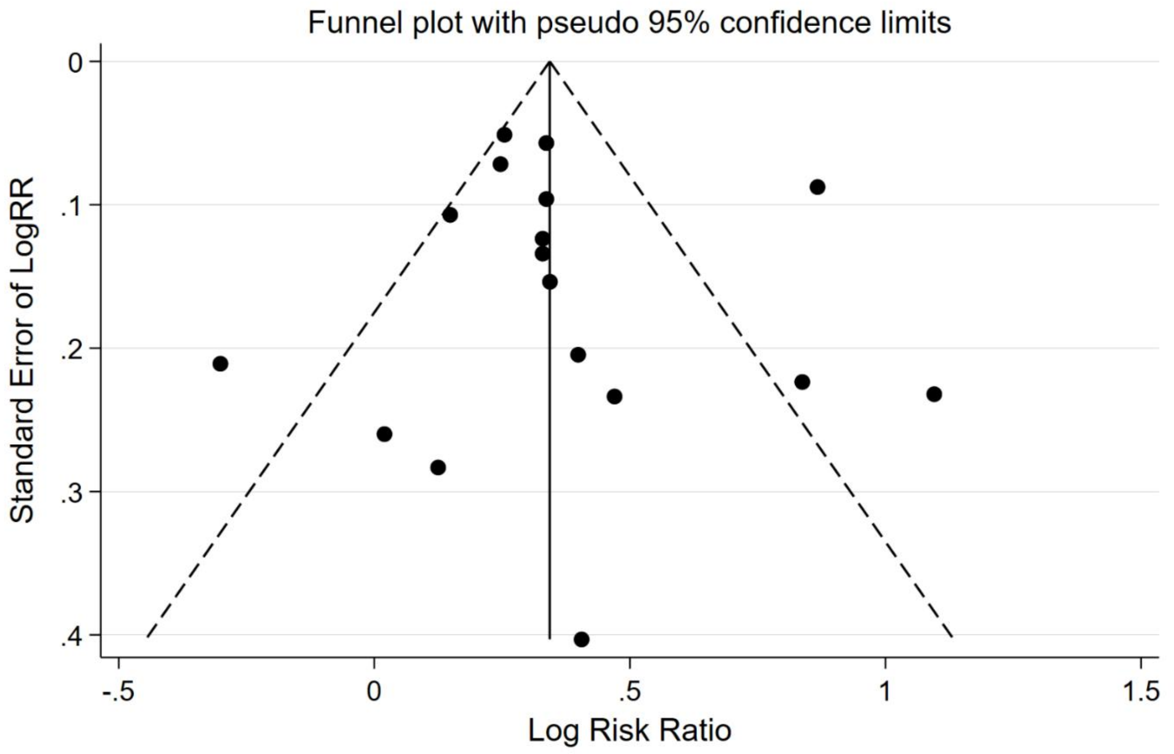

Figure S11. Funnel plot to detect publication bias for ≥4 ACEs vs 0 ACEs and risk of diabetes, Egger test, P=0.669

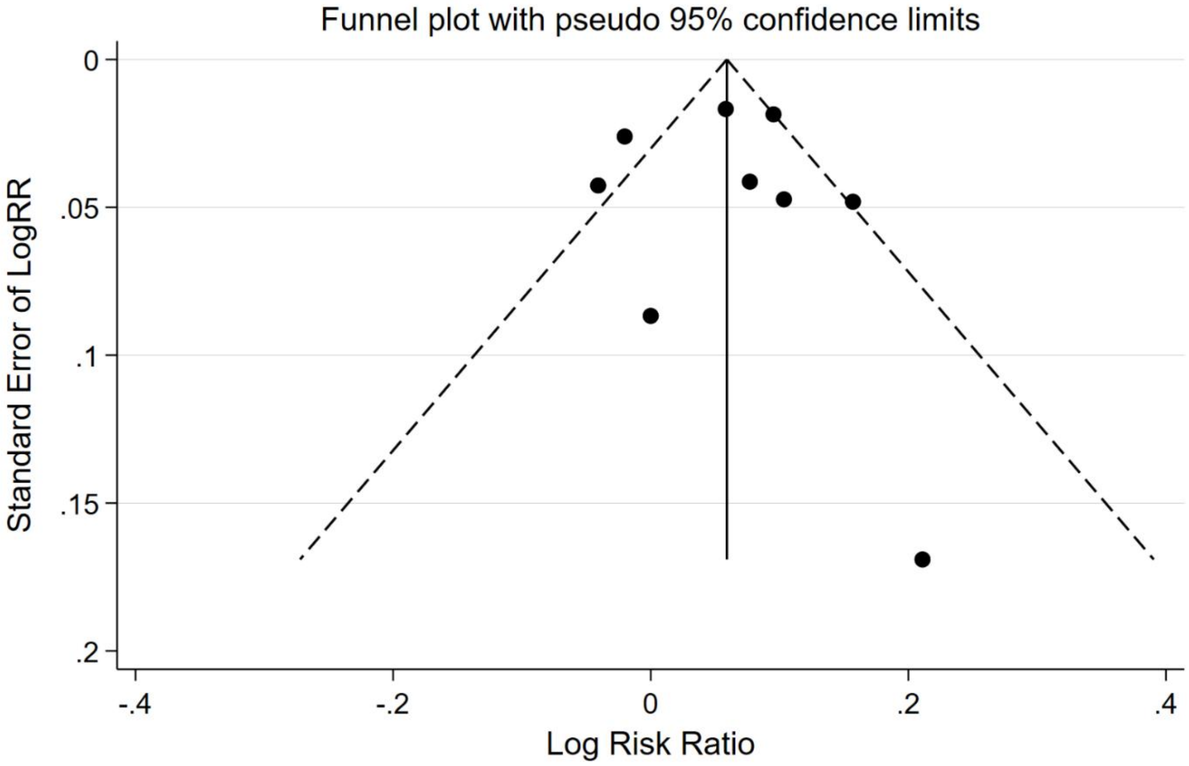

Figure S12. Funnel plot to detect publication bias for continuous ACEs and risk of diabetes

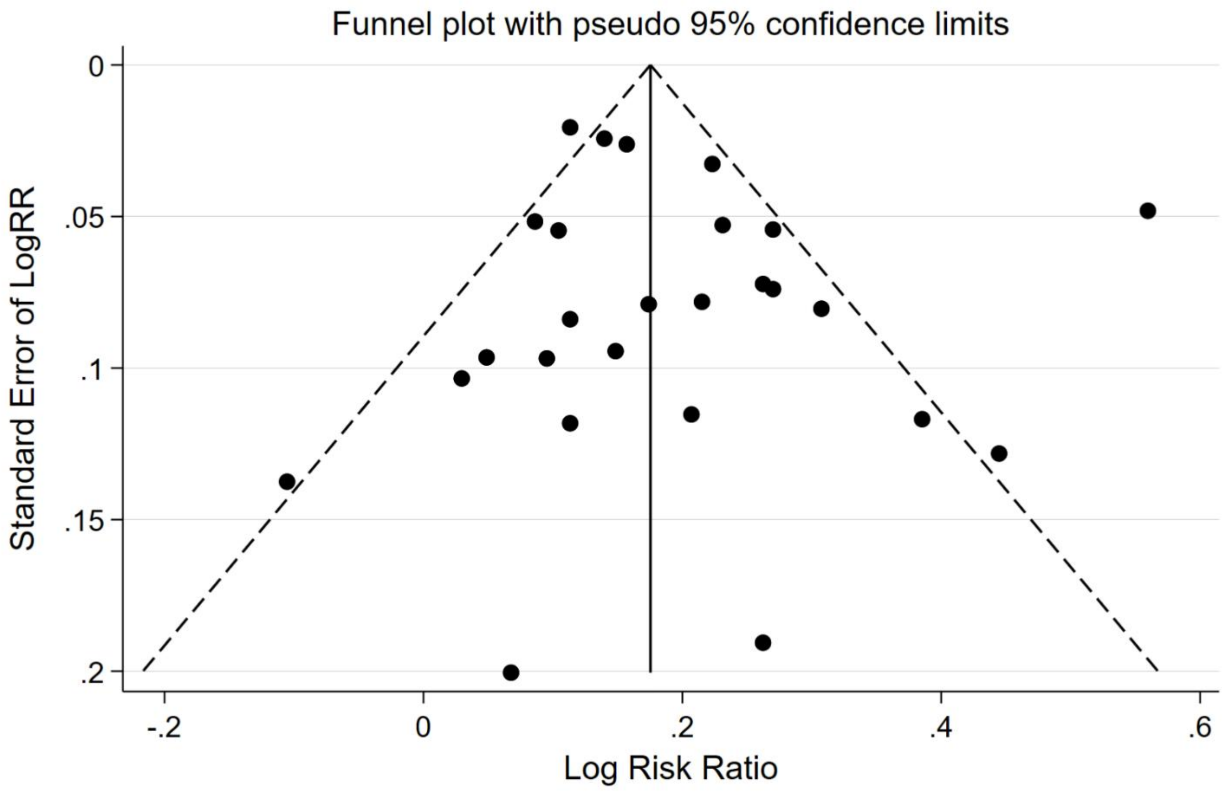

Figure S13. Funnel plot to detect publication bias for any ACE vs 0 ACEs and risk of diabetes, Egger test, P=0.362

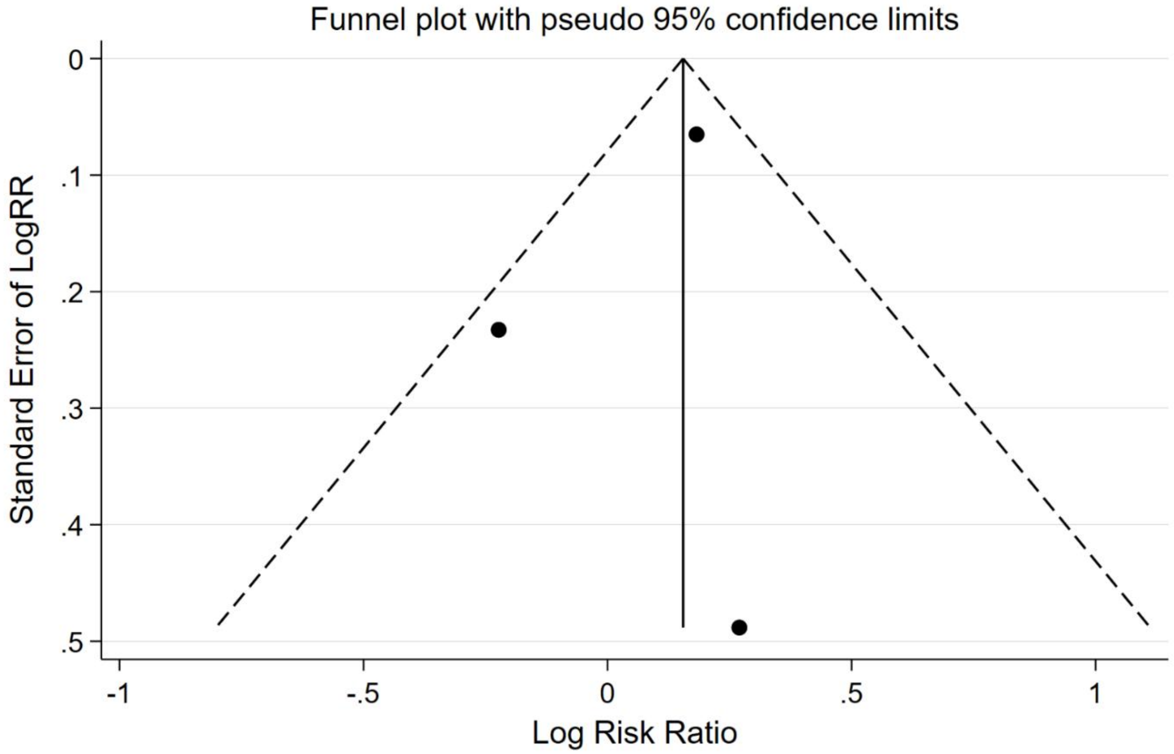

Figure S14. Funnel plot to detect publication bias for ≥4 ACEs vs <4 ACEs and risk of diabetes

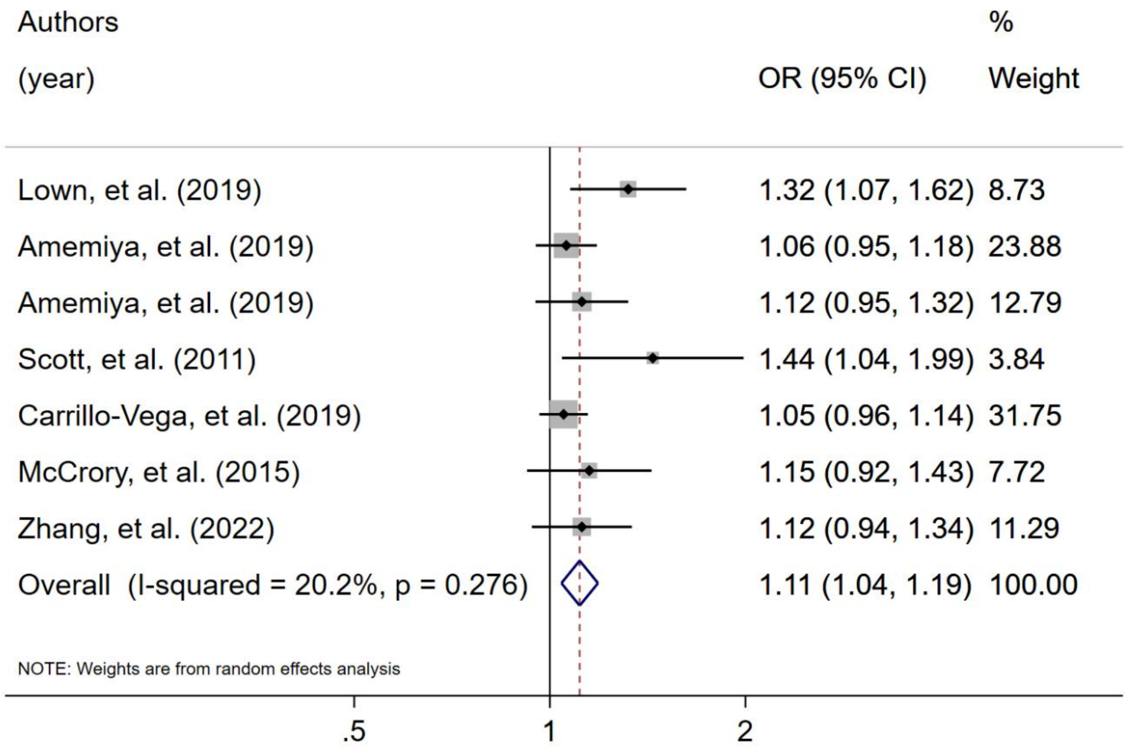

Figure S15. Forest plot for economic adversity and risk of diabetes, Reference: none

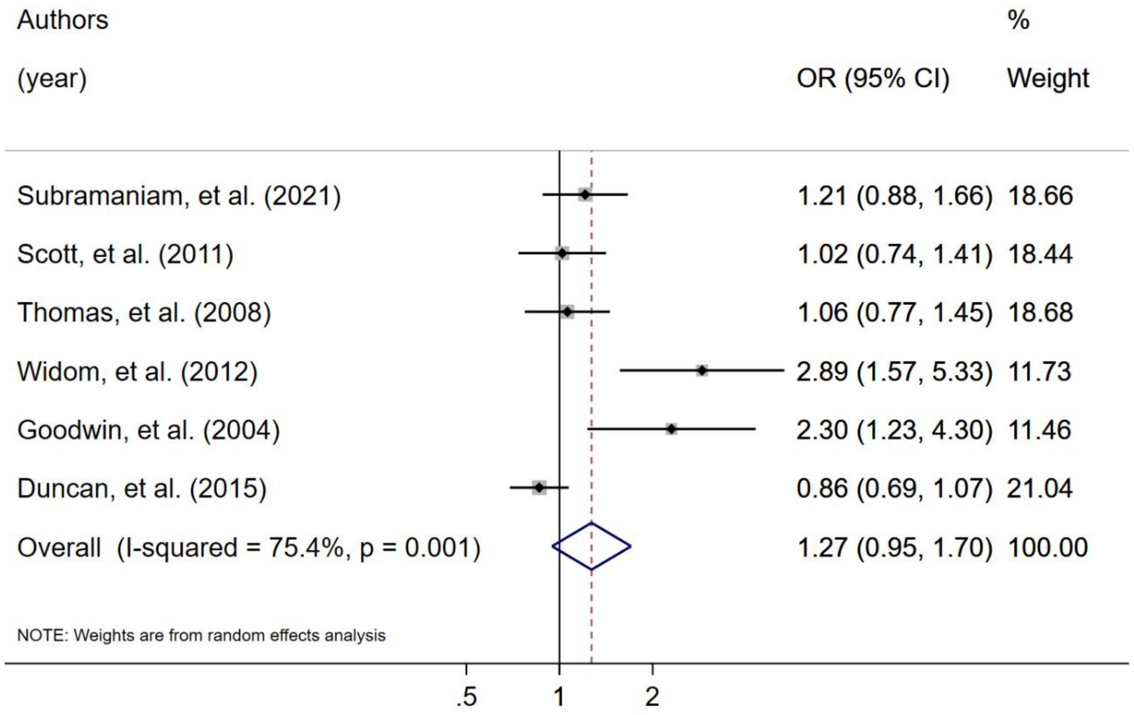

Figure S16. Forest plot for neglect and risk of diabetes, Reference: none

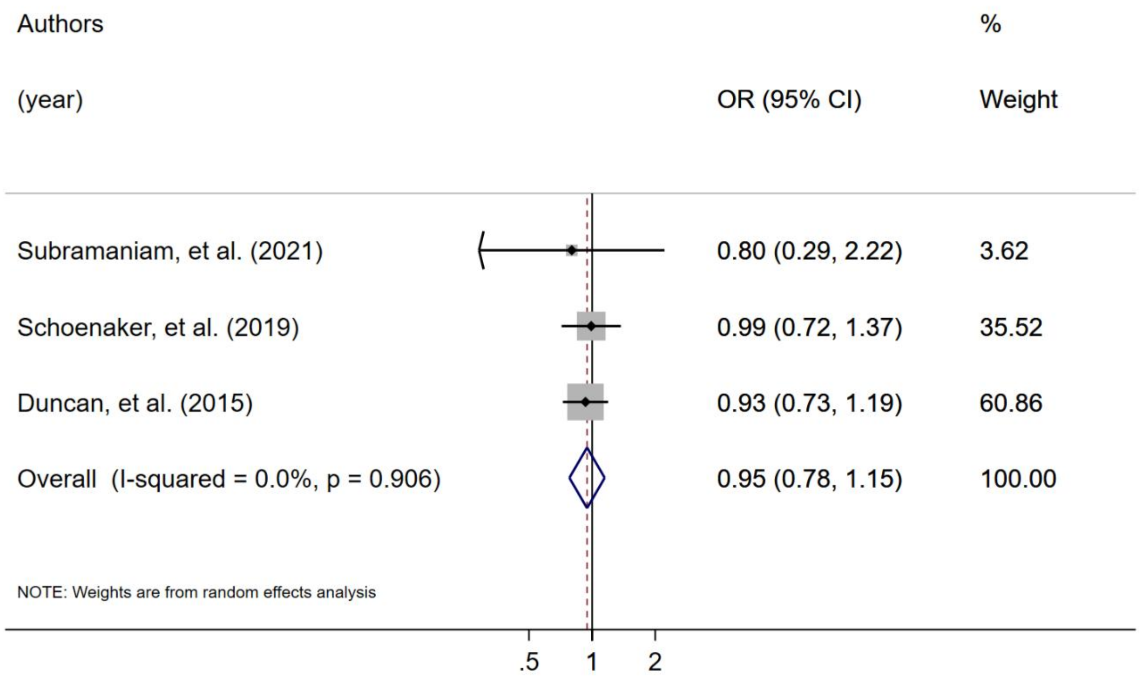

Figure S17. Forest plot for emotional abuse and risk of diabetes, Reference: none

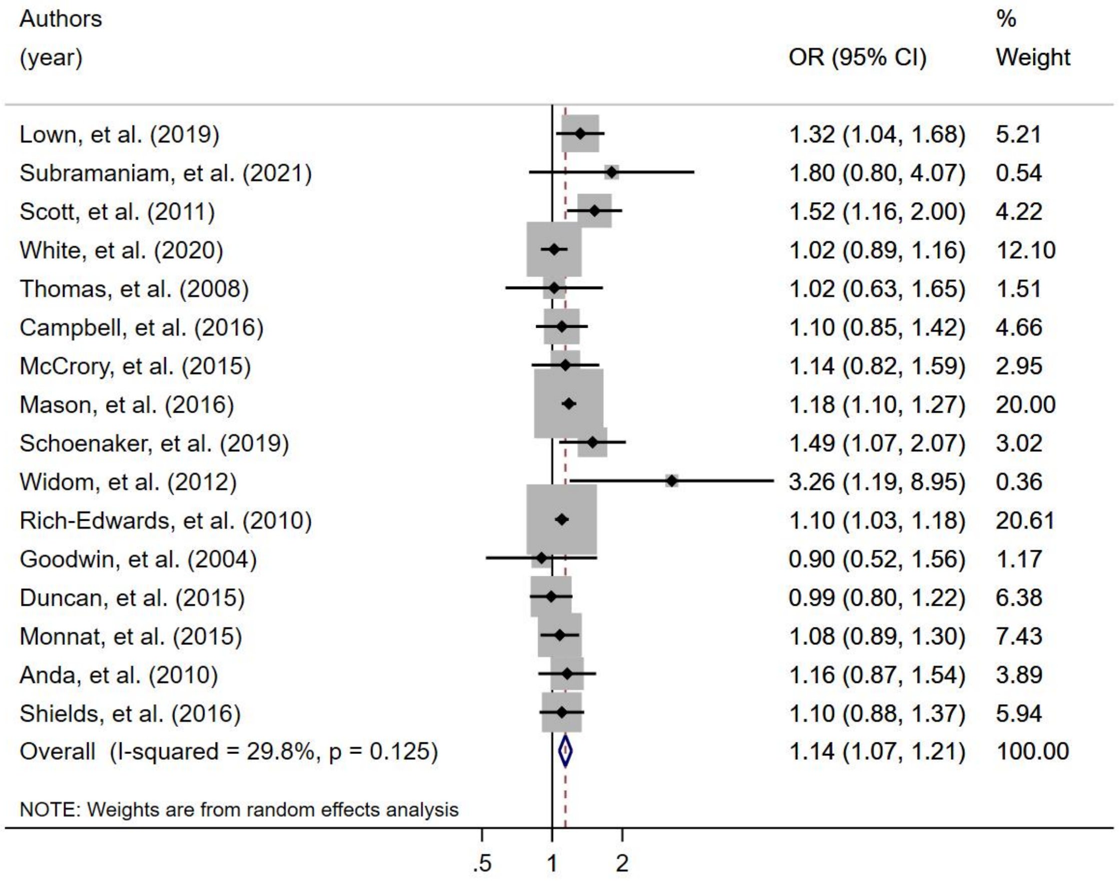

Figure S18. Forest plot for physical abuse and risk of diabetes, Reference: none

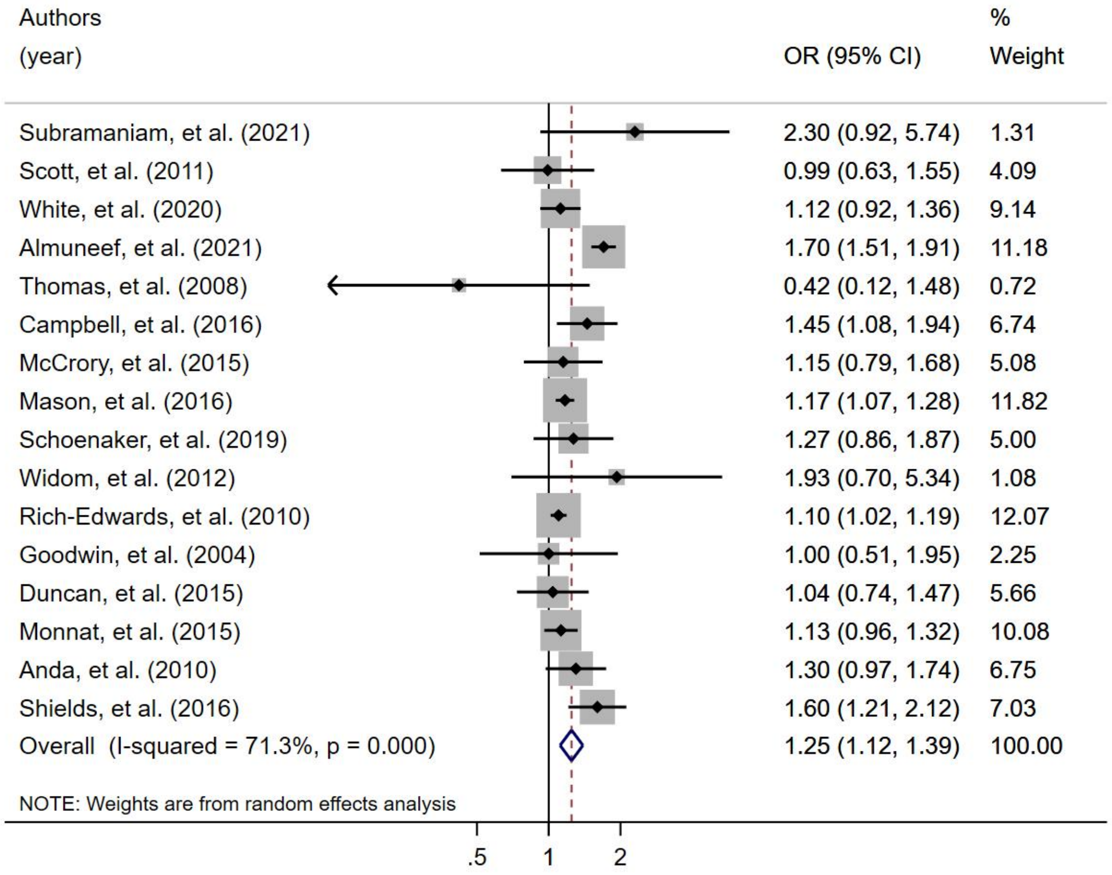

Figure S19. Forest plot for sexual abuse and risk of diabetes, Reference: none

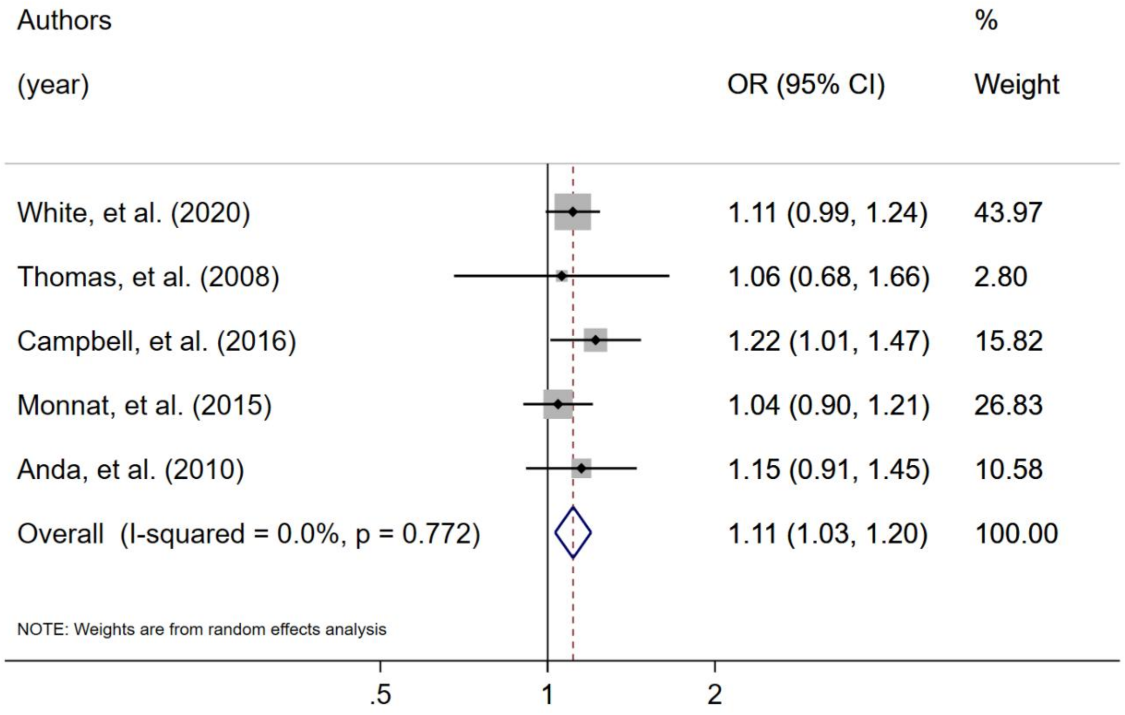

Figure S20. Forest plot for verbal abuse and risk of diabetes, Reference: none

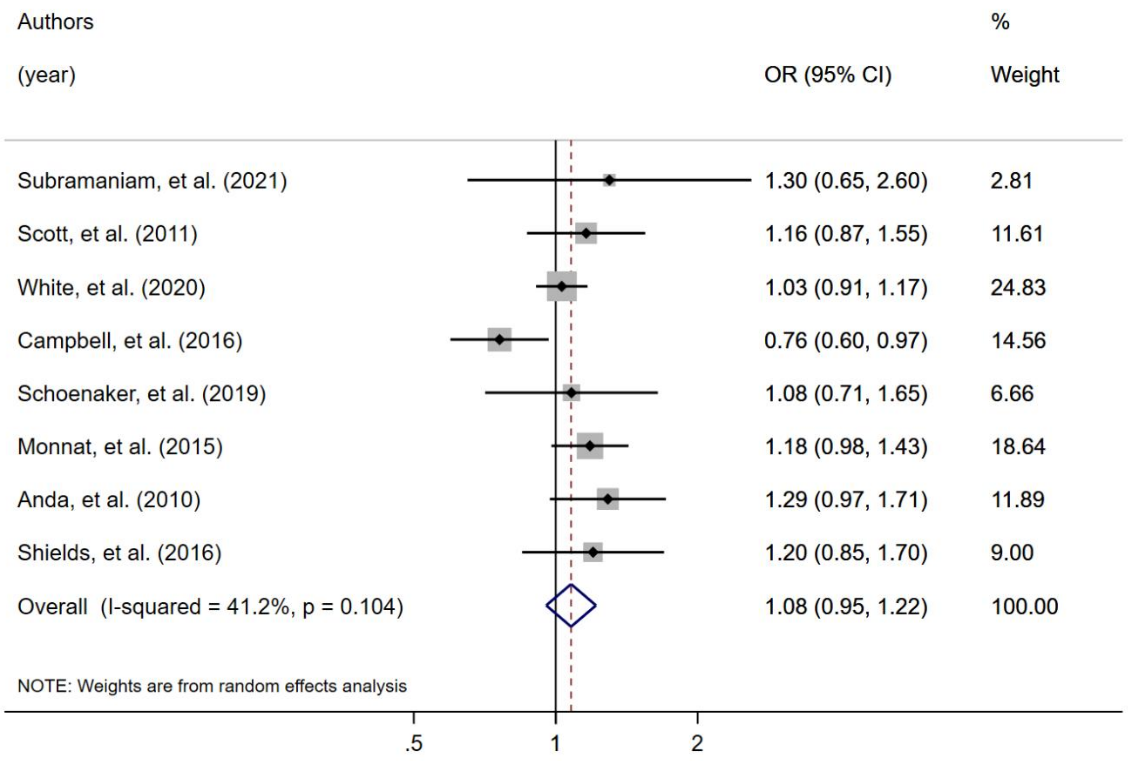

Figure S21. Forest plot for domestic violence and risk of diabetes, Reference: none

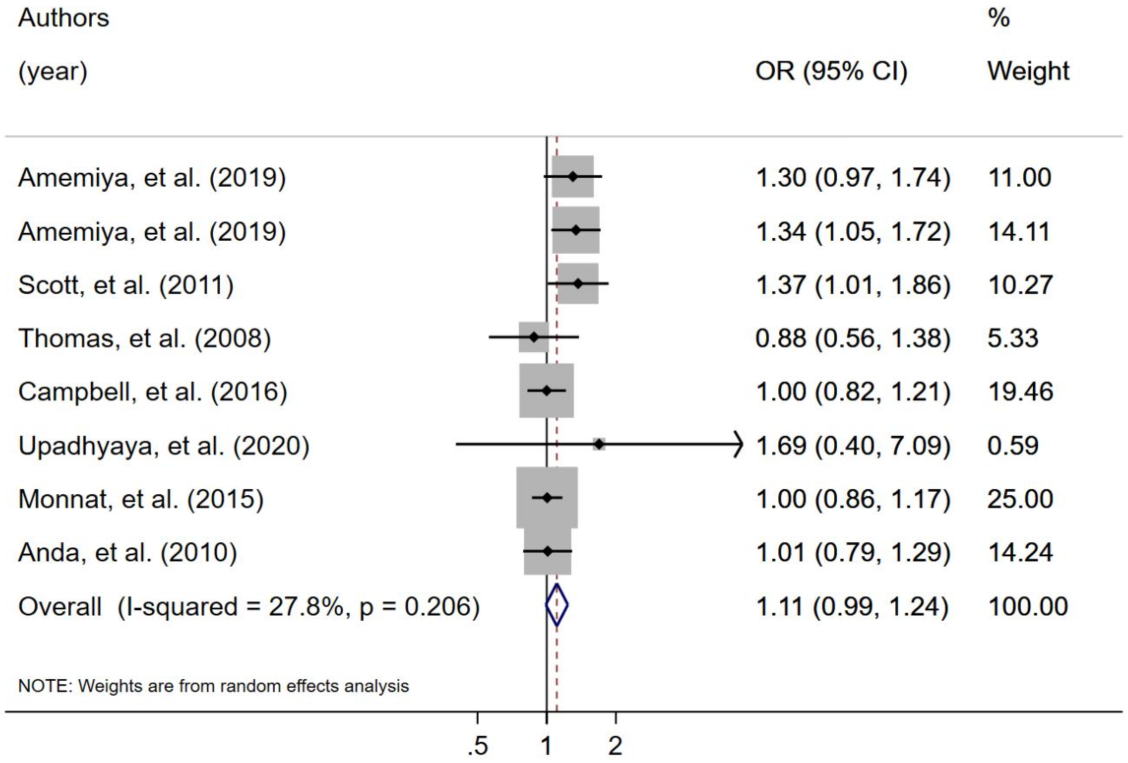

Figure S22. Forest plot for parental divorce/separation and risk of diabetes, Reference: none

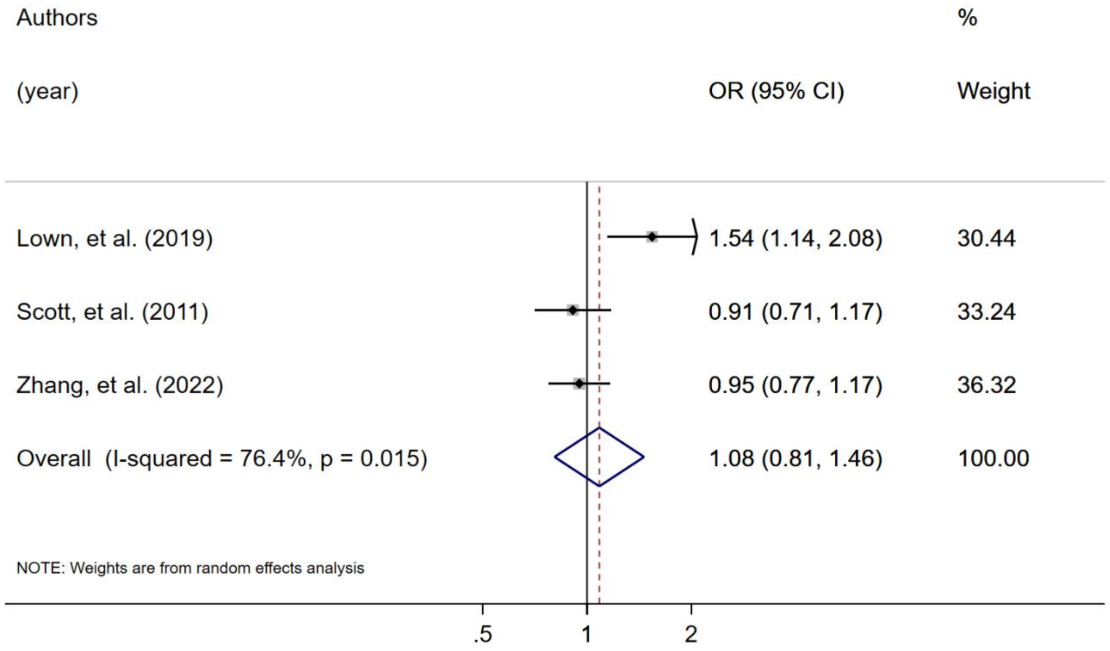

Figure S23. Forest plot for parental death and risk of diabetes, Reference: none

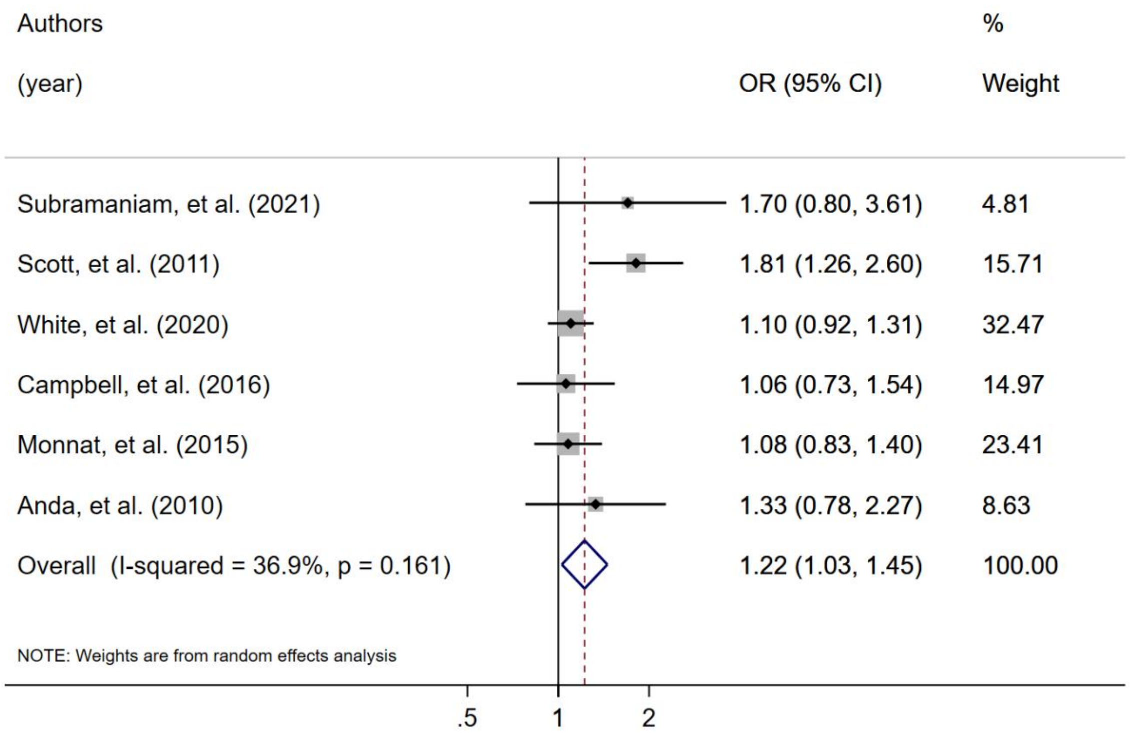

Figure S24. Forest plot for incarceration and risk of diabetes, Reference: none

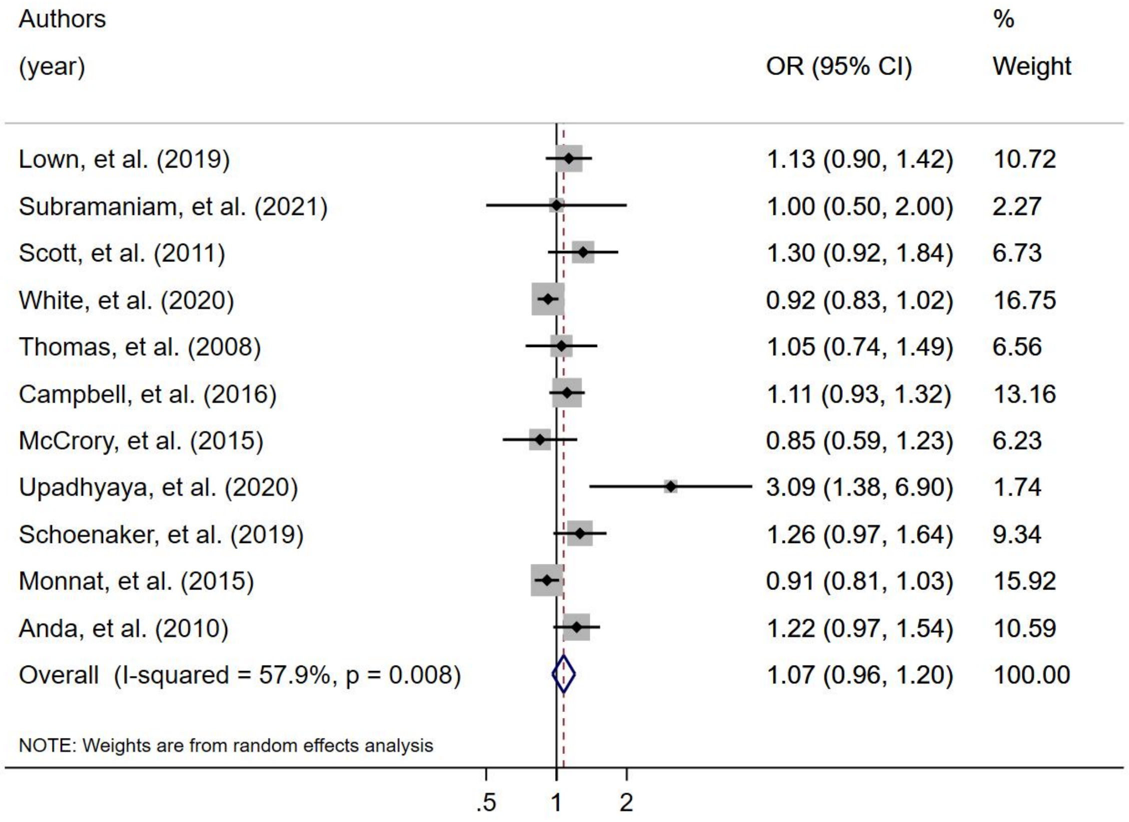

Figure S25. Forest plot for family member with substance abuse and risk of diabetes, Reference: none

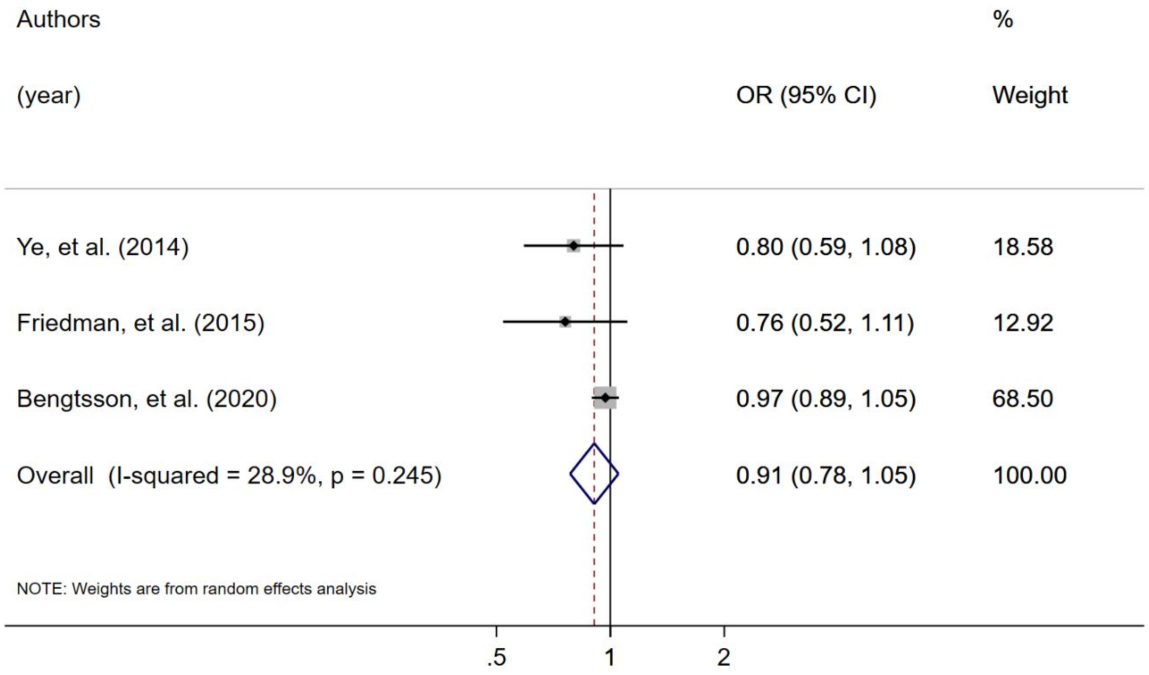

Figure S26. Forest plot for family member with substance abuse and risk of diabetes, Reference: no ACEs

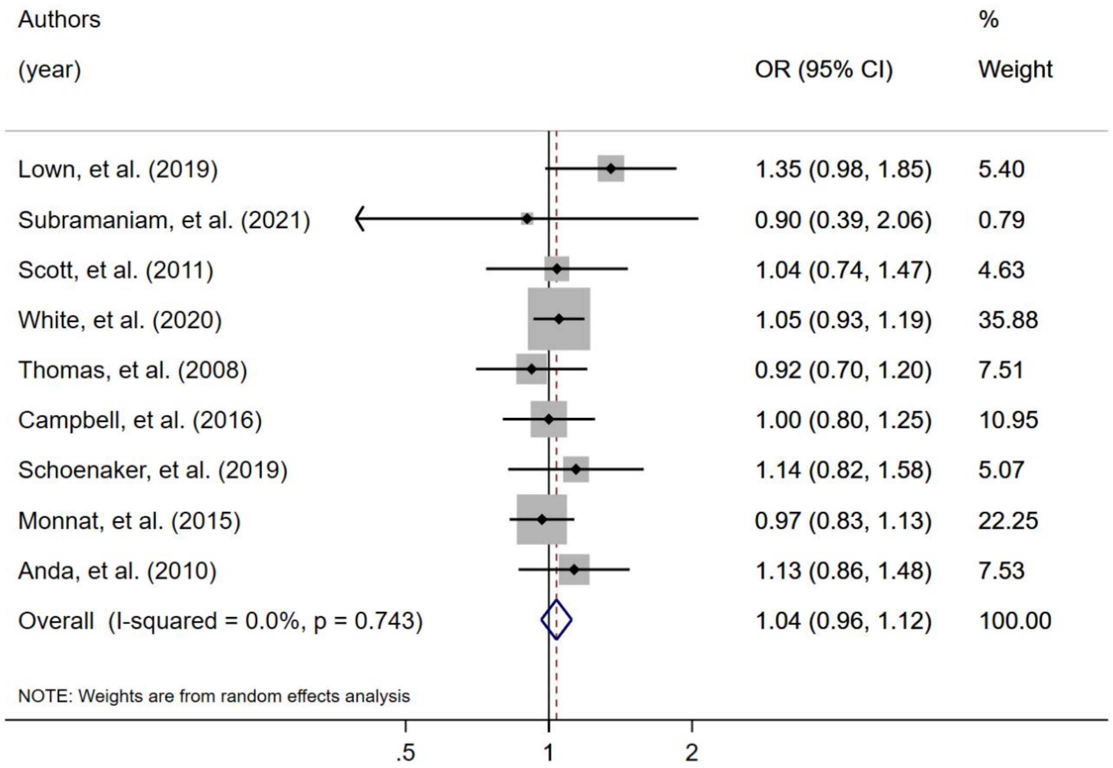

Figure S27. Forest plot for family member with mental disorder and risk of diabetes, Reference: none

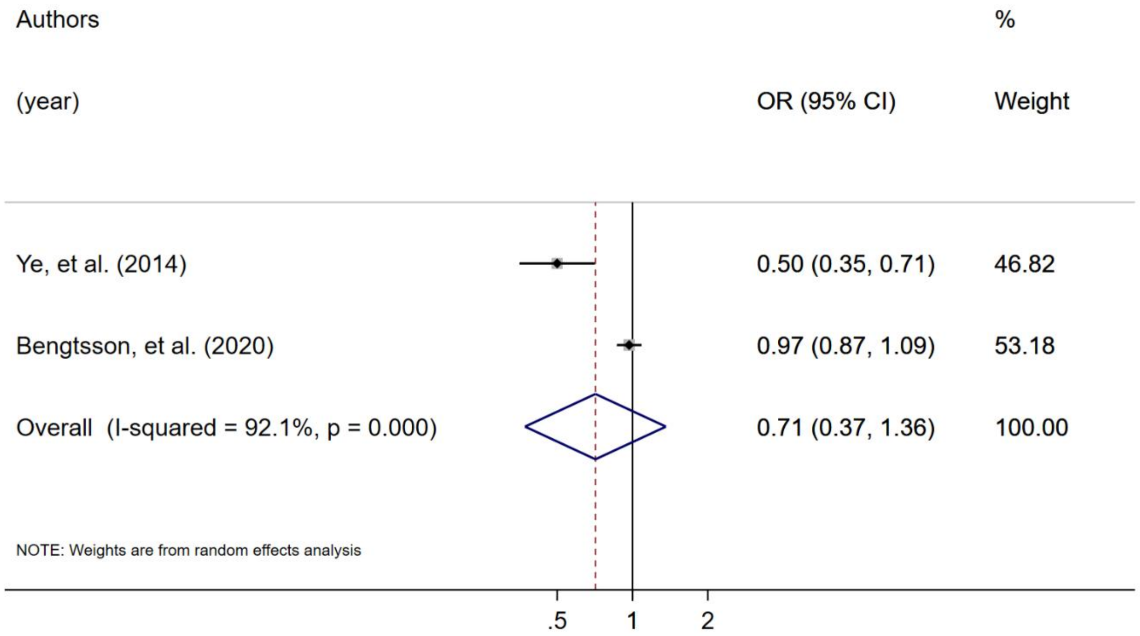

Figure S28. Forest plot for family member with mental disorder and risk of diabetes, Reference: no ACEs

References

1. Goodwin RD, Stein MB. Association between childhood trauma and physical disorders among adults in the United States. Psychological Medicine. 2004;34(3):509-520.
2. Thomas C, Hyppönen E, Power C. Obesity and type 2 diabetes risk in midadult life: The role of childhood adversity. Pediatrics. 2008;121(5):e1240-e1249.
3. Alastalo H, Raikkonen K, Pesonen AK, Osmond C, Barker DJ, Kajantie E, et al. Cardiovascular health of Finnish war evacuees 60 years later. Annals of Medicine. 2009;41(1):66-72.
4. Ramiro LS, Madrid BJ, Brown DW. Adverse childhood experiences (ACE) and health-risk behaviors among adults in a developing country setting. Child Abuse & Neglect. 2010;34(11):842-855.
5. Rich-Edwards JW, Spiegelman D, Lividoti Hibert EN, Jun HJ, Todd TJ, Kawachi I, et al. Abuse in childhood and adolescence as a predictor of type 2 diabetes in adult women. American Journal of Preventive Medicine. 2010;39(6):529-536.
6. Anda RF. Adverse childhood experiences and population health in Washington. 2010.
7. Scott KM, Von Korff M, Angermeyer MC, Benjet C, Bruffaerts R, De Girolamo G, et al. Association of childhood adversities and early-onset mental disorders with adult-onset chronic physical conditions. Archives of General Psychiatry. 2011;68(8):838-844.
8. Widom CS, Czaja SJ, Bentley T, Johnson MS. A prospective investigation of physical health outcomes in abused and neglected children: New findings from a 30-year follow-up. American Journal of Public Health. 2012;102(6):1135-1144.
9. Lynch L, Waite R, Davey MP. Adverse childhood experiences and diabetes in adulthood: Support for a collaborative approach to primary care. Contemporary Family Therapy. 2013;35(4):639-655.
10. Ye D, Reyes-Salvail F. Adverse childhood experiences among Hawai'i adults: Findings from the 2010 Behavioral Risk Factor Survey. Hawai'i Journal of Medicine & Public Health. 2014;73(6):181-190.
11. McCauley HL, Blosnich JR, Dichter ME. Adverse childhood experiences and adult health outcomes among veteran and non-veteran women. Journal of Women's Health. 2015;24(9):723-729.
12. Gilbert LK, Breiding MJ, Merrick MT, Thompson WW, Ford DC, Dhingra SS, et al. Childhood adversity and adult chronic disease: An update from ten states and the District of Columbia, 2010. American Journal of Preventive Medicine. 2015;48(3):345-349.
13. McCrory C, Dooley C, Layte R, Kenny RA. The lasting legacy of childhood adversity for disease risk in later life. Health Psychology. 2015;34(7):687-696.

14. Bellis MA, Hughes K, Leckenby N, Hardcastle KA, Perkins C, Lowey H. Measuring mortality and the burden of adult disease associated with adverse childhood experiences in England: A national survey. Journal of Public Health. 2015;37(3):445-454.
15. Friedman EM, Montez JK, Sheehan CM, Guenewald TL, Seeman TE. Childhood adversities and adult cardiometabolic health: Does the quantity, timing, and type of adversity matter? Journal of Aging and Health. 2015;27(8):1311-1338.
16. Duncan AE, Auslander WF, Bucholz KK, Hudson DL, Stein RI, White NH. Relationship between abuse and neglect in childhood and diabetes in adulthood: Differential effects by sex, national longitudinal study of adolescent health. Preventing Chronic Disease. 2015;12:E70.
17. Monnat SM, Chandler RF. Long term physical health consequences of adverse childhood experiences. The Sociological Quarterly. 2015;56(4):723-752.
18. Almuneef M, Hollinshead D, Saleheen H, AlMadani S, Derkash B, AlBuhairan F, et al. Adverse childhood experiences and association with health, mental health, and risky behavior in the kingdom of Saudi Arabia. Child Abuse & Neglect. 2016;60:10-17.
19. Campbell JA, Walker RJ, Egede LE. Associations between adverse childhood experiences, high-risk behaviors, and morbidity in adulthood. American Journal of Preventive Medicine. 2016;50(3):344-352.
20. Wade R, Jr., Cronholm PF, Fein JA, Forke CM, Davis MB, Harkins-Schwarz M, et al. Household and community-level adverse childhood experiences and adult health outcomes in a diverse urban population. Child abuse & neglect. 2016;52:135-145.
21. Mason SM, Tobias DK, Clark CJ, Zhang C, Hu FB, Rich-Edwards JW. Abuse in childhood or adolescence and gestational diabetes: A retrospective cohort study. American Journal of Preventive Medicine. 2016;50(4):436-444.
22. Ford K, Butler N, Hughes K, Quigg Z, Bellis MA, Barker P. Adverse childhood experiences (ACEs) in Hertfordshire, Luton and Northamptonshire. Liverpool: Liverpool John Moores University. 2016.
23. Shields ME, Hovdestad WE, Pelletier C, Dykxhoorn JL, O'Donnell SC, Tonmyr L. Childhood maltreatment as a risk factor for diabetes: Findings from a population-based survey of Canadian adults. BMC Public Health. 2016;16(1):1-12.
24. Wade R, Jr., Becker BD, Bevans KB, Ford DC, Forrest CB. Development and evaluation of a short adverse childhood experiences measure. American Journal of Preventive Medicine. 2017;52(2):163-172.
25. Llabre MM, Schneiderman N, Gallo LC, Arguelles W, Daviglus ML, Gonzalez F, 2nd, et al. Childhood trauma and adult risk factors and disease in Hispanics/Latinos in the US: Results from the Hispanic Community Health Study/Study of Latinos (HCHS/SOL) Sociocultural Ancillary Study. Psychosomatic Medicine. 2017;79(2):172.
26. Chanlongbutra A, Singh GK, Mueller CD. Adverse childhood experiences, health-related quality of life, and chronic disease risks in rural areas of the United States. Journal of Environmental and Public Health. 2018;2018:7151297.

27. Lown EA, Lui CK, Karriker-Jaffe K, Mulia N, Williams E, Ye Y, et al. Adverse childhood events and risk of diabetes onset in the 1979 National longitudinal survey of youth cohort. BMC Public Health. 2019;19(1):1-13.
28. Amemiya A, Fujiwara T, Shirai K, Kondo K, Oksanen T, Pentti J, et al. Association between adverse childhood experiences and adult diseases in older adults: A comparative cross-sectional study in Japan and Finland. BMJ Open. 2019;9(8):e024609.
29. Carrillo-Vega MF, Albavera-Hernández C, Ramírez-Aldana R, García-Peña C. Impact of social disadvantages in the presence of diabetes at old age. BMC Public Health. 2019;19(1):1-10.
30. Felitti VJ, Anda RF, Nordenberg D, Williamson DF, Spitz AM, Edwards V, et al. Relationship of childhood abuse and household dysfunction to many of the leading causes of death in adults: The adverse childhood experiences (ACE) study. American Journal of Preventive Medicine. 2019;56(6), 774-786.
31. Merrick MT, Ford DC, Ports KA, Guinn AS, Chen J, Klevens J, et al. Vital signs: Estimated proportion of adult health problems attributable to adverse childhood experiences and implications for prevention—25 States, 2015–2017. Morbidity and Mortality Weekly Report. 2019;68(44):999-1005.
32. Kreatsoulas C, Fleegler EW, Kubzansky LD, McGorrian CM, Subramanian SV. Young adults and adverse childhood events: A potent measure of cardiovascular risk. The American Journal of Medicine. 2019;132(5):605-613.
33. Salas J, van den Berk-Clark C, Skiöld-Hanlin S, Schneider FD, Scherrer JF. Adverse childhood experiences, depression, and cardiometabolic disease in a nationally representative sample. Journal of Psychosomatic Research. 2019;127:109842.
34. Schoenaker D, Callaway LK, Mishra GD. The role of childhood adversity in the development of gestational diabetes. American Journal of Preventive Medicine. 2019;57(3):302-310.
35. White BA, West KJ, Fuller-Thomson E. Is exposure to family member incarceration during childhood linked to diabetes in adulthood? Findings from a representative community sample. SAGE Open Medicine. 2020;8:2050312120905165.
36. El Mhamdi S, Lemieux A, Ben Fredj M, Bouanene I, Ben Salah A, Abroug H, et al. Social and early life adversity and chronic health conditions among Tunisian adults. Translational Behavioral Medicine. 2020;10(4):949-958.
37. Ittoop T, Jeffrey K, Cheng CI, Reddy S. The relationship between adverse childhood experiences and diabetes in central Michigan adults. Endocrine Practice. 2020;26(12):1425-1434.
38. Stanhope KK, Cammack AL, Perreira KM, Fernández-Rhodes L, Cordero C, Gallo LC, et al. Adverse childhood experiences and lifetime adverse maternal outcomes (gestational diabetes and hypertensive disorders of pregnancy) in the Hispanic Community Health Study/Study of Latinos. Annals of epidemiology. 2020;50:1-6.

39. Upadhyaya S, Tolmunen T, Elomaa AP, Ruohomäki A, Kraav SL, Kauhanen L, et al. Parental alcohol problems, parental divorce, and type 2 diabetes in adulthood: A longitudinal prospective cohort study in middle-aged men. Psychosomatic Medicine. 2020;82(9):817-822.
40. Flores-Torres MH, Comerford E, Signorello L, Grodstein F, Lopez-Ridaura R, de Castro F, et al. Impact of adverse childhood experiences on cardiovascular disease risk factors in adulthood among Mexican women. Child Abuse & Neglect. 2020;99:104175.
41. Bengtsson J, Byberg S, Carstensen B, De Stavola BL, Svensson J, Jørgensen ME, et al. Accumulation of childhood adversities and type 1 diabetes risk: a register-based cohort study of all children born in Denmark between 1980 and 2015. International Journal of Epidemiology. 2020;49(5):1604-1613.
42. Waehrer GM, Miller TR, Silverio Marques SC, Oh DL, Burke Harris N. Disease burden of adverse childhood experiences across 14 states. PLoS One. 2020;15(1):e0226134.
43. Bala K, Monteiro K, Kole-White M, Gjelsvik A, High P. The association between adverse childhood experiences and diabetes status during pregnancy among women in Rhode Island, 2016-2018. Rhode Island Medical Journal. 2020;103(9):52-55.
44. Subramaniam M, Abdin E, Vaingankar JA, Chang S, Sambasivam R, Jeyagurunathan A, et al. Association of adverse childhood experiences with diabetes in adulthood: Results of a cross-sectional epidemiological survey in Singapore. BMJ Open. 2021;11(3):e045167.
45. Almuneef M. Long term consequences of child sexual abuse in Saudi Arabia: A report from national study. Child Abuse & Neglect. 2021;116:103967.
46. Versteegen M, Bozlak CT, Larkin H, Appleton AA. Maternal depression, adverse childhood experiences, and social support in relation to gestational diabetes risk: Results from the Albany Infant and Mother Study (AIMS). BMC Pregnancy and Childbirth. 2021;21(1):1-7.
47. Lin L, Wang HH, Lu C, Chen W, Guo VY. Adverse childhood experiences and subsequent chronic diseases among middle-aged or older adults in China and associations with demographic and socioeconomic characteristics. JAMA Network Open. 2021;4(10):e2130143.
48. Zhang K, Wu B, Zhang W. Adverse childhood experiences in relation to comorbid cardiovascular diseases and diabetes among middle-aged and old adults in China. Geriatrics & Gerontology International. 2022;22(1):12-18.
